# A nematic liquid crystal elastomer rotary engine

**DOI:** 10.1038/s41598-025-33311-0

**Published:** 2025-12-23

**Authors:** Takuya Ohzono, Hirohmi Watanabe, Eugene M. Terentjev

**Affiliations:** 1 Core Electronics Technology Research Institute, National Institute of Advanced Industrial Science and Technology (AIST), 1-1-1 Higashi, Tsukuba, 305-8565 Japan; 2 Clare Hall, University of Cambridge, Herschel Rd, Cambridge, CB3 9AL UK; 3 Research Institute for Sustainable Chemistry, AIST, 3-11-32 Kagami-yama, Higashi-Hiroshima, Hiroshima 739-0046 Japan; 4 Cavendish Laboratory, University of Cambridge, J.J. Thomson Avenue, Cambridge, CB3 0HE UK

**Keywords:** Liquid crystal elastomer, Rubber engine, Photo-thermal energy, Viscoelasticity, Engineering, Materials science, Physics

## Abstract

Nematic liquid crystal elastomers (NLCEs) could perform as soft actuators because they generate greater contraction stress than ordinary rubber in response to a temperature rise of several tens of Kelvin. In this study, we follow the Wiegand rubber motor design, and develop a prototype star-shaped type rotary engine with four to twelve NLCE contractile elements, in which multiple NLCE actuators contract due to the photo-thermal effect to rotate the crankshaft. Continuous rotational motion under white light was experimentally confirmed. Through theoretical modeling, it was clarified that in this system, the contraction of the NLCE actuators generates a driving torque, while a viscoelasticity-originated internal friction resists the rotation, thus producing rotation at constant speed. Effects of the number of NLCE elements, light power, initially applied pre-tension, and the system size on the engine performances, such as rotation speed, output power, energy efficiency, are explored. Given that the physical properties of NLCE (liquid crystallinity, phase transition temperature, and viscoelastic properties) are linked to the engine performance, the present engine system with the analysis protocol is expected to serve as a test platform for future evaluation of NLCE actuation performance.

**Supplementary Information:**

The online version contains supplementary material available at 10.1038/s41598-025-33311-0.

## Introduction

Soft actuator materials are key to realizing soft robotics, flexible medical devices and dynamic structures, particularly at small scales, where hard actuators cannot be applied. Such materials are often stimulus-sensitive soft polymeric materials with shape morphing and stress generation capabilities. One of the candidates, studied for many years, is the nematic liquid crystal elastomer (NLCE)^1–4^, which can undergo much greater deformation and stress than conventional rubbers^5^ in response to the temperature change and other stimuli. This significant mechanical change occurs due to the underlying phase transition between the nematic liquid crystalline phase and the isotropic phase. This transition is accompanied by a substantial change in the mechanical properties of the material, often characterized by the tensile stress-strain response. At high temperatures, in the isotropic phase, the material exhibits the ordinary entropy-based rubber elasticity, whereas in the nematic phase at low temperatures, a very soft strain region called ‘soft elasticity’ appears^1^. This is due to the entropic elasticity being cancelled by the rotating axis of orientational order of the nematic phase, adapting the local orientation to the imposed strain^6^. The stress-strain curve shape changes significantly between the two phases. For example, if strain is kept constant, the stress changes significantly^7,8^. If the stress is kept constant, the length changes significantly, allowing work to be extracted externally^3,7^.

To drive actuation, or the stress generation in NLCEs, crossing the nematic-isotropic phase transition is essential, and the most straightforward external stimulus for this is heat. Another well-studied approach involves using light^9–15^, affecting nematic order by either photo-isomerization or a direct light-induced local heating, offering spatiotemporal actuation controls. The NLCE systems capable of inducing the high-speed deformation of materials using light have also been reported^16^. An example of a rotational motor that performs continuous motion under steady light irradiation has also been reported by Ikeda et al.^17^, although their design was based on bending and can produce no useful work. Related theoretical studies exploring different possible designs of a continuous-motion rotary motors^18,19^ made progress in identifying possible mechanical systems to realize the steady motion. To date, however, no study has been conducted that systematically examines the NLCE-based engine system and provides a theoretical analysis based on experimental results.

The mechanism of an engine made of ordinary rubber bands, which are similar to NLCE polymers other than in liquid crystallinity, has been known for a long time and was mainly considered as educational toys for understanding thermodynamical cycles. These are known as “rubber engines”. Strong has reviewed^20^ rubber engines that convert slight changes in the entropic rubber elasticity on temperature changes into rotational motion. These are interesting examples of thermodynamic discussion on heat engines, such as the Carnot cycle, being realized in solids. However, their poor performance made them unsuitable for practical use. The main reason for this is that the mechanical properties of rubbers do not change enough on temperature change. Consequently, the potential for applications could instead be realized through a rubber engine utilizing an NLCE with their higher driving power.

The central question guiding the present study is the impact of replacing the ordinary rubber with NLCE on the operation of the rubber engine, and the subsequent enhancement in performance that would bring. This system is therefore called the NLCE engine. We experimentally study one of the NLCE rotary engines, with a theoretical discussion examining its operation. Basic properties of the present NLCE are first characterized, including the nematic-isotropic transition temperature and stress-strain response. The simplified model is formulated to help us understand the driving mechanism, mechanical properties and issues of energy efficiency, with most of the details given in Supplementary Information. The model incorporates the viscoelasticity of NLCE, a feature that is fundamental in these elastomers, importantly leading to an enhanced damping within the nematic phase. Finally, we demonstrate the model star-shaped NLCE rotary engine, and characterize its rotation at various values of operation parameters, including the initially applied pre-tension to each NLCE actuating element, the number of such elements around the ring, and the applied light power.

## Results and discussion

**Fig. 1:**
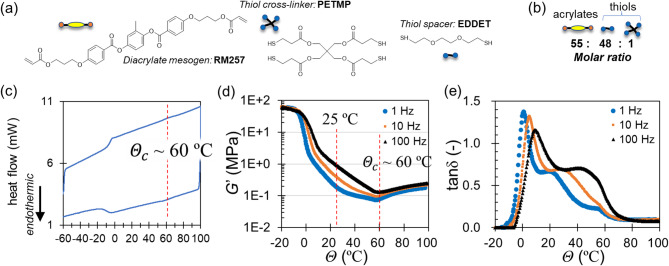
Basic properties of NLCE. (**a**) Chemical formulae of the components of the traditional thiol-acrylate main-chain NLCE. (**b**) Molar ratio of polymer units, defining the crosslinking density (or equivalently: the average length of anisotropic network strands). (**c**) DSC thermograms illustrating the glass and nematic-isotropic transitions. Dynamic-mechanical test results on: the storage shear modulus *G`* (**d**) and the loss factor tanδ (**e**) on sample cooling at 5 °C/min.

###  Basic properties of NLCE

 First, the basic physical properties of the NLCE used in this study are described. The main-chain thiol-acrylate NLCE prepared via the well-known thiol-ene click reaction^21,22^ of monomers with the molar ratio (see Methods, Fig. 1a, b) shows the nematic phase at room temperature. The thermal analysis of the NLCE using differential scanning calorimetry (DSC) (Fig. 1c) showed the nematic–isotropic transition temperatures ($\it \:{\varTheta\:}_{c}$) around 60°C, which is a temperature that can be easily exceeded by light irradiation, making it possible to induce a phase transition for driving the engine by white light for local heating. The dynamic mechanical analysis (DMA) results (Fig. 1d, e), showing a characteristic kink in *G’* around the phase transition around 60 °C, also showing the growth in the modulus and low damping in the isotropic phase. It can also be seen that in the nematic state at temperatures below $\it \:{\varTheta\:}_{c}$, including room temperature, the loss factor tanδ, is high, suggesting that the effective viscosity is high, see for detail^3^.

**Fig. 2:**
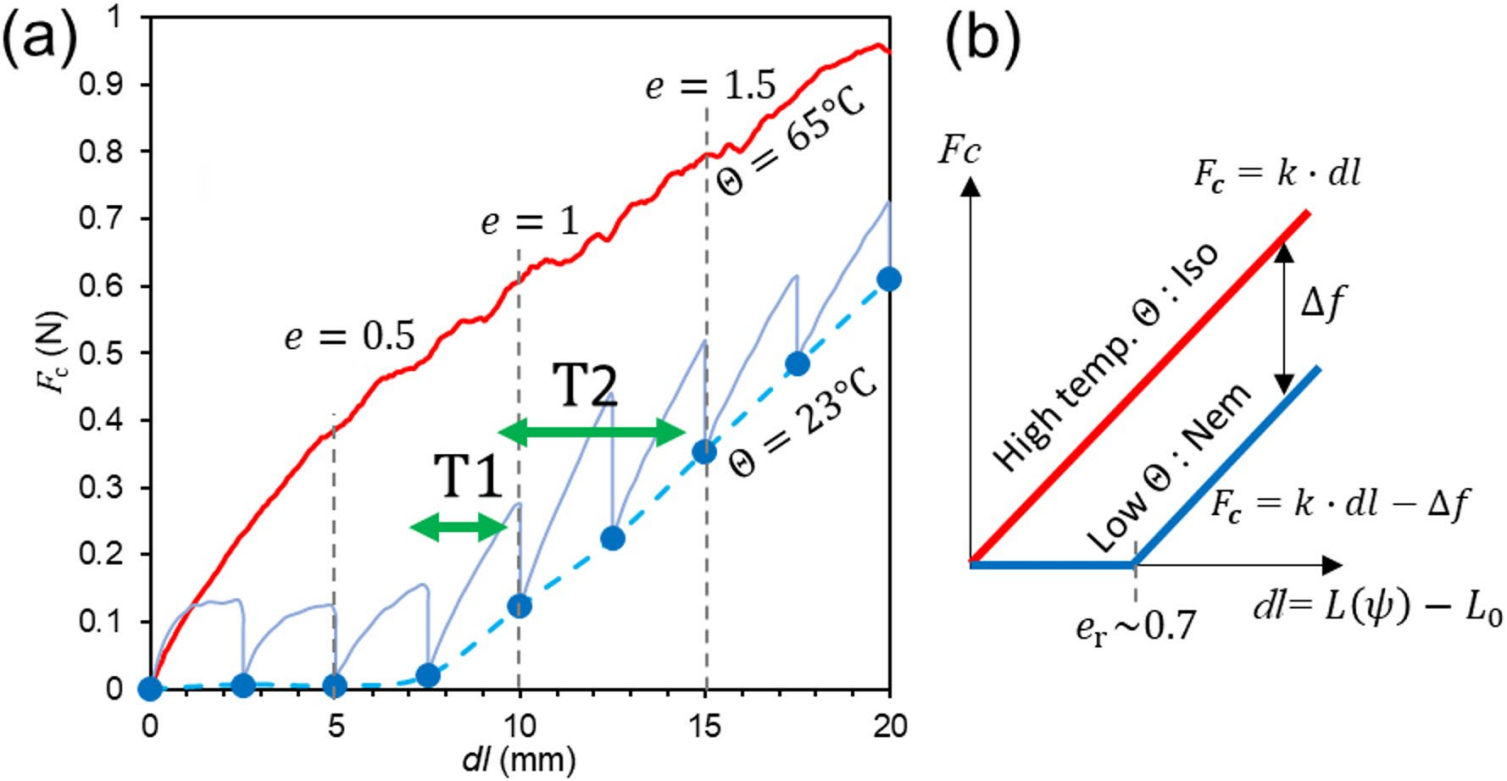
Force-extension curves of NLCE. (**a**) Experimental results with the NLCE strip with the initial thickness of 0.5 mm, length $\:{L}_{0}$ of 10 mm, and width of 5 mm. The usual entropic elasticity is observed at higher temperature, $\it \:{\varTheta\:>\varTheta\:}_{c}$. The soft-elastic response with the soft strain range of $\:{e}_{\mathrm{r}}\sim0.7$ with slow stress relaxation at in the nematic phase is confirmed. The strain changes on engine operations under different pre-strain cases, T1 and T2, are shown. (**b**) The model force-extension relations used in the theoretical model.

The force ($\:{F}_{\boldsymbol{c}}$ )-extension (*dl*) curves at high and low temperatures are shown on Fig. 2a. As shown in the previous studies^23–27^, relaxation is very slow in the nematic phase due to anomalously high internal friction of NLCE. Thus, the stress-strain (or force-extension) curve presented in Fig. 2a is obtained by allowing 300 s of stress relaxation for each strain step is shown for the ’low *Θ*’ curve. For the high temperature curve in the isotropic phase, the result obtained with the continuous stretching at the rate of 0.00083 s^-1^ is shown. The significant difference between these curves is the reason why NLCE is performing as a cyclic soft actuator in our motor. This is evident when the curves are compared with the corresponding changes in the natural rubber curves due to temperature changes (see Supplemental Fig. S1), which were measured for reference. In the case of natural rubber, the change is based solely on temperature-dependent rubber modulus and the associated change in stress (or force) when the temperature rises of ~ 30 K at a constant strain of *e* = 0.5 is approximately + 5%~0.1 N. In contrast, the stress change at a constant strain of *e* = 1 in this NLCE is + 600%~0.5 N (Fig. 2a, note that the stress at *e* = 0.5 is zero in NLCE on the soft elastic plateau). This large difference originates from the soft elasticity at lower strains in the nematic phase, which is the unique characteristics of NLCE^1,24^.

For theoretical model to be discussed later, we simplify the observed stress-strain curves with simple linear functions (Fig. 2b). Although the experimental curves are highly nonlinear in the nematic regime, it is considered as a linear spring *k* as a first approximation. The low temperature curve, $\:{F}_{\boldsymbol{c}}=k\cdot\:dl-\varDelta\:f$ is the combination of the zero-stress range at lower strains, which corresponds to the soft elastic range, and a linear spring with the identical slope to that, $\:{F}_{\boldsymbol{c}}=k\cdot\:dl$, at high temperature^28^. The specific function system will be described in the modeling section later.

The DMA results shown in Fig. 1d, e also suggest that the viscoelastic relaxation at low temperature in the nematic may affect the dynamics of the engine. The rotation of the wheel in the engine is accompanied by the continuous expansion and contraction process of NLCE strips, which occurs on a time scale that is proportional to its rotational speed. In this cycle, the contraction power stroke occurs at elevated temperatures, while elongation occurs at reduced temperatures. It is therefore expected that the long viscoelastic relaxation that is exhibited during the latter elongation step of the cycle will resist the engine operation, which is an undesirable effect in general mechanical systems.

This negative effect was previously often disregarded in simple rubber engines where the internal friction is relatively low; however, recent advancements now permit their evaluation in NLCE engines for the first time. The positive effect on engine operation resulting from the mentioned stress-strain curve difference will be considered, balanced against the negative effect arising from this viscoelastic relaxation. In our simplified model, the effect of internal friction is incorporated as the angular-velocity-dependent friction term with a constant friction coefficient as a first approximation. More precisely, we should consider the friction coefficient that varies with time and temperature rather than the constant one. However, we will leave this more complex approach for the future and instead adopt the model in its simplest form, which allows us to account for the effects of the internal friction in the actuator that had not been considered before.

**Fig. 3:**
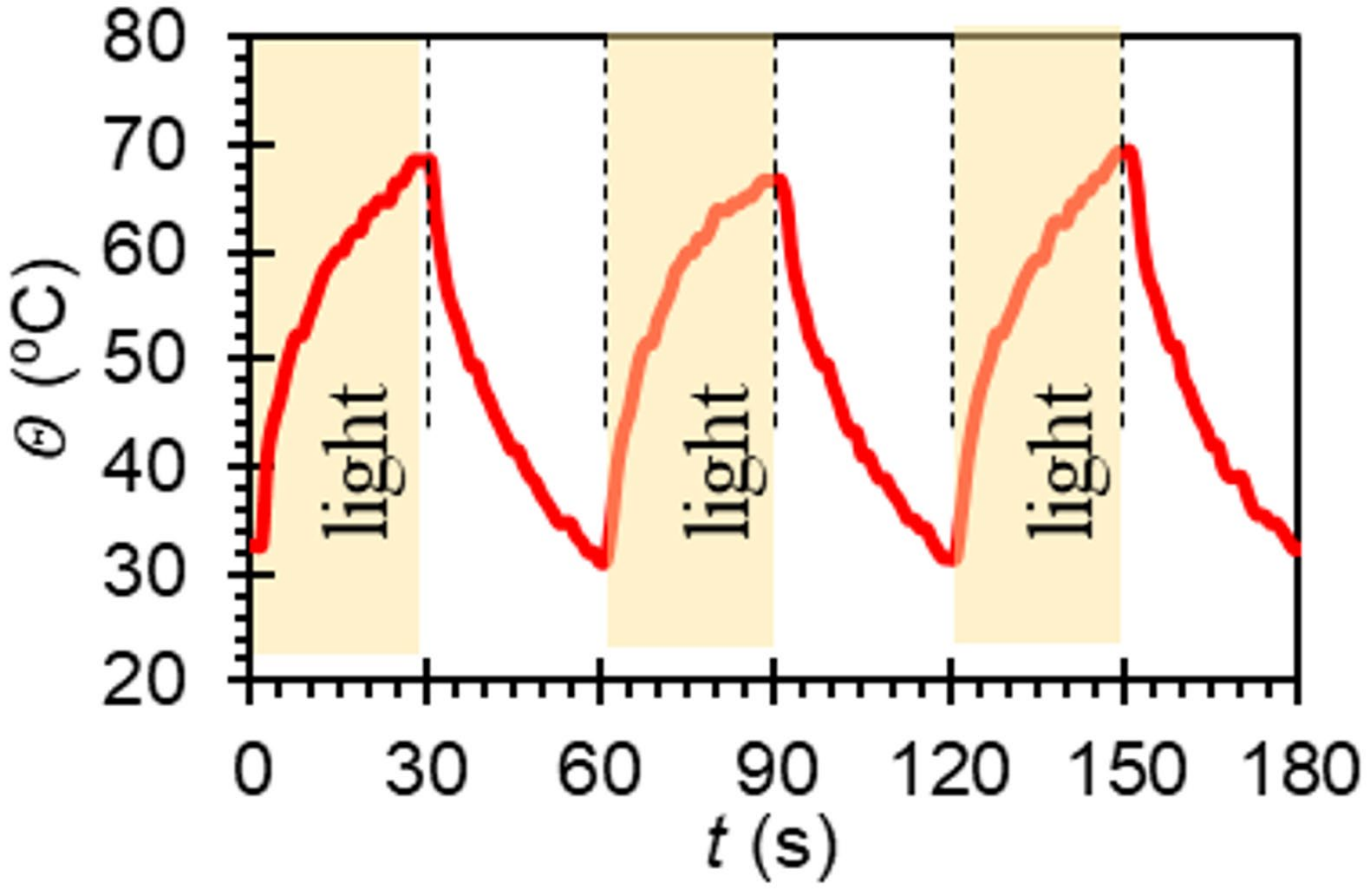
Typical temperature response of NLCE to white light irradiation. The typical change in temperature upon white light irradiation at a room temperature (25 °C).

Our way to impart light-sensitivity on NLCE is simple black paint, which causes local heating on exposure to light. The typical change in temperature achieved in this way upon white light irradiation at room temperature (25 °C) is shown in Fig. 3. It is clear that both heating and ambient un-assisted cooling take time, but the heating time required to reach the phase transition temperature $\:{\it \varTheta\:}_{c}\sim60\:$°C will vary depending on the light power (the natural cooling will always be slow). In the following theoretical model, this temperature change is greatly simplified, assuming that the temperature changes instantaneously and remains at a constant high temperature during light irradiation and at a low temperature during non-irradiation.

###  NLCE rotary engine design

**Fig. 4:**
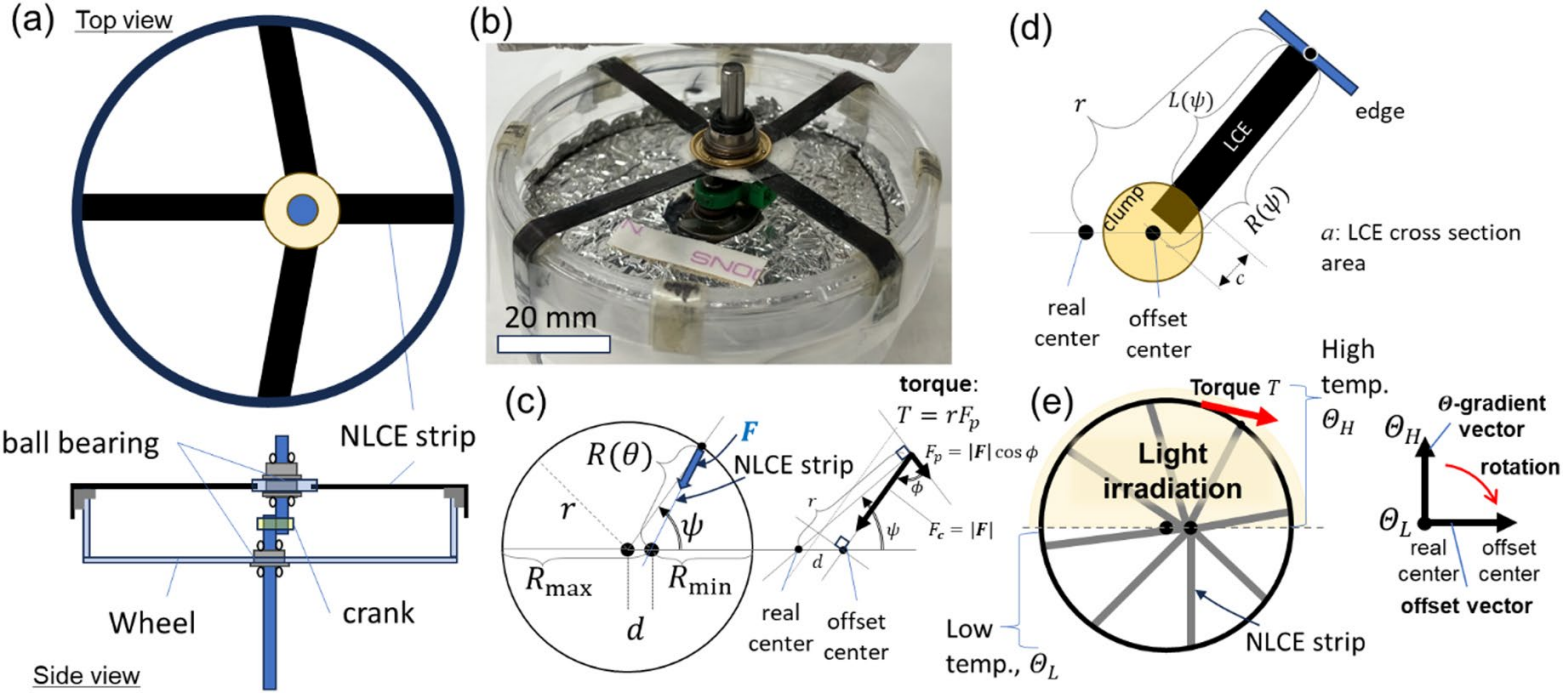
NLCE rotary engine design. (**a**) Top and side views. The case with *N* = 4 is shown. (**b**) Actual photograph. (**c**) Some geometrical definitions of the system for the single NLCE case. $\:R\left(\psi\:\right)$ changes with when revolution, further changing the tensile force on NLCE, $\:{F}_{\boldsymbol{c}}$, producing torque on the main wheel from the NLCE. The net torque is the sum of the multiple NLCE strips. (**d**) A more realistic schematic diagram of an installed NLCE strip. (**e**) The area of light irradiation to produce the temperature gradient in the system (*N* = 8). The rotation direction can be expected from the temperature gradient vector and the offset vector.

 The present engine design (Fig. 4a-b) is based on the Wiegand rubber engine that is one of engines reviewed by Strong^20^. In the rubber engine, the net force generated by the contraction of rubber bands mounted radially around the wheel is converted into rotational torque on the wheel via the crankshaft mechanism^29^, producing rotational motion (Fig. 4c). In this design, heating a portion of the rubber bands creates a non-zero net torque $\:T$ due to the crankshaft misalignment with respect to the rotational center, causing the wheel to rotate with the bands (Fig. 4c-d). As the heating position is fixed, the rubber band to be heated changes in sequence, one after another, as the wheel rotates (Fig. 4e). The steady rotation is created by causing multiple power strokes by rubber bands with staggered timing. This mechanism is identical to common internal combustion engines and, especially regarding the radial configuration of driving units (rubber bands here) to the star-shaped engine commonly found in propeller-driven airplanes.

In an internal combustion engine, the power stroke involves a rapid increase in internal pressure due to the rise in temperature caused by fuel combustion in the engine cylinders. The volume change that drives the cam of the crankshaft is based on a change in gas pressure. In a rubber engine, however, the contraction of stretched rubber due to heating produces the power stroke, which is based on a temperature-dependent change in rubber elasticity. Therefore, although both engine types depend on the power stroke based on entropy change, the difference lies in the state of the substance that produces it: gas (fuel) or solid (rubber).

In the Wiegand rubber engine^20^, the heat is transferred to the rubber under pre-tension, causing it to contract. In our motor, the temperature rise is induced by the light absorption in the black painted NLCE strips installed on the half side of the wheel. Other mechanical systems are essentially the same as those of the Wiegand-type^20^.

### Theoretical modeling

 For convenience, the physical parameters used in this study (Supplemental Note 1 and 2) are listed in Table 1. The main theoretical equations and derived relations regarding the engine dynamics and energy balance (Supplemental Note 1 and 2) are listed in Table 2.

**Table 1:**
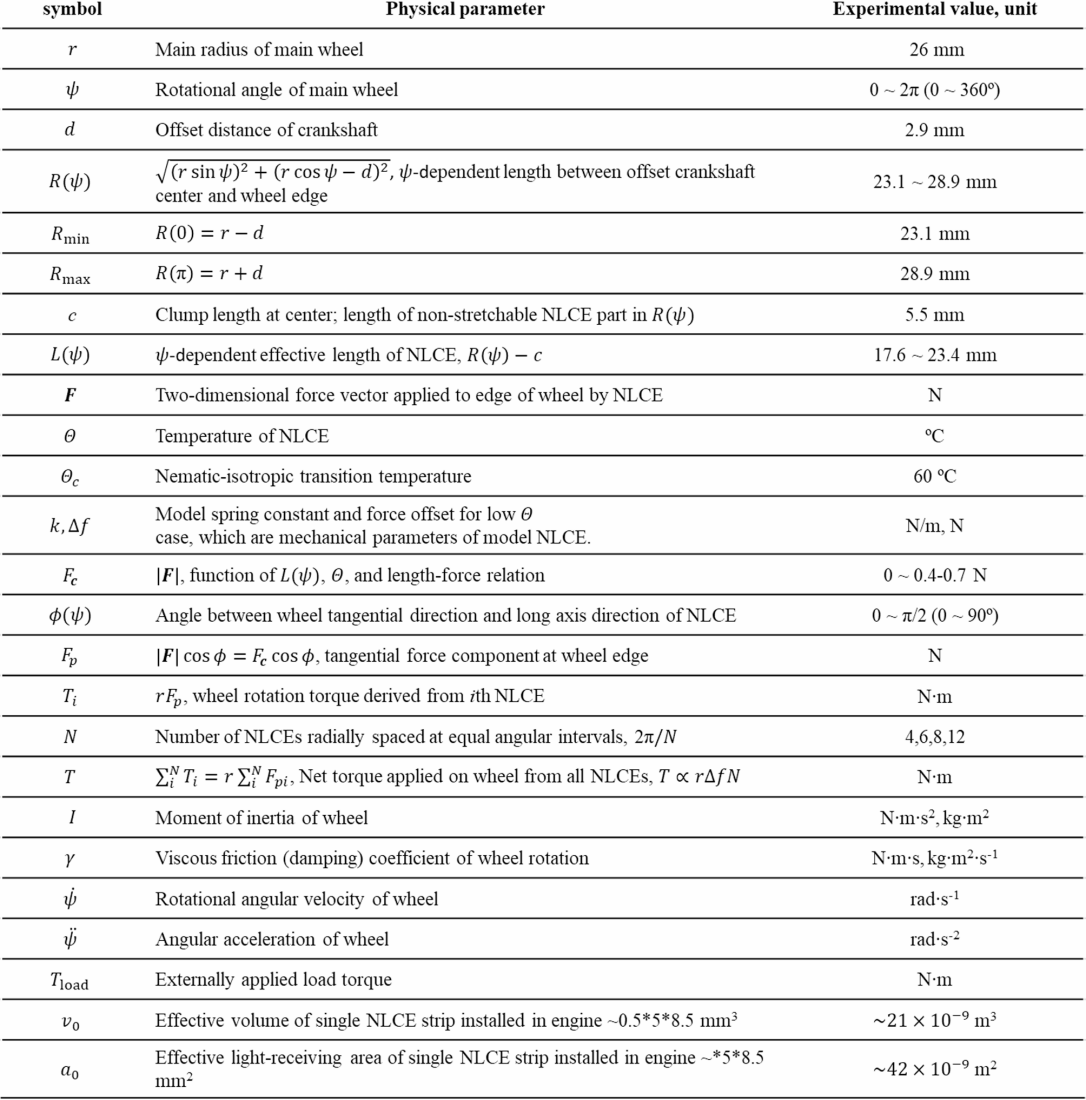
List of parameters. See supplemental notes 1 and 2 for detailed derivations.

**Table 2:**
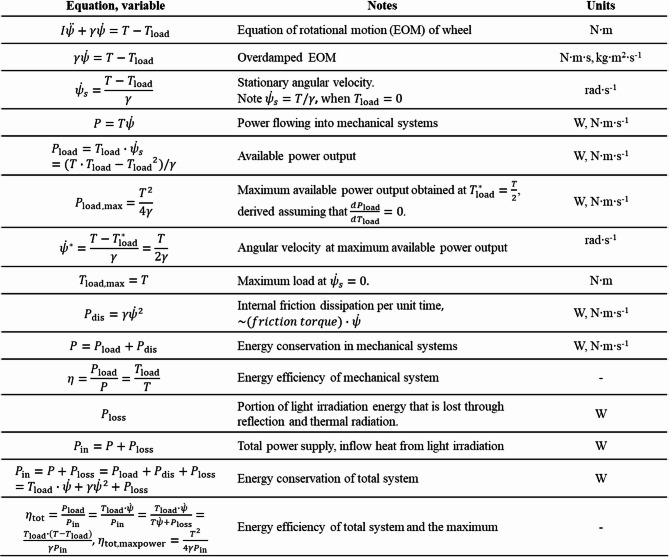
The list of key relations connecting the engine dynamics and energy balance. See supplemental notes 1–3 for detailed derivations.

The detailed derivation of the present theoretical model is described in Supplemental Note 1 with Supplemental Figs. S2 and S3, while here only the key conclusions relevant to the experimental results are noted. When the number of NLCE strips $\:N$ is large, a net torque $\:T$ is generated under partial light irradiation (Fig. 4e and Supplemental Fig. S4) regardless of the rotation angle $\:\psi\:$, which can be expressed as $T\sim r\Delta fN/\pi \propto r\Delta fN$, where $\:\varDelta\:f$ can be modulated by light power and/or initially applied strain, and $\:N$ can be changed when NLCE strips are installed. Given $\:T$, the rotational dynamics can be simply discussed via the rotational equation of motion of the main wheel, $\:I\ddot{\psi\:}+\gamma\:\dot{\psi\:}=T-{T}_{\mathrm{l}\mathrm{o}\mathrm{a}\mathrm{d}}$, where the angular-velocity-dependent linear rotational friction of the system is apparently introduced (Supplemental Fig. S4). Here, the system friction coefficient, $\:\gamma\:=N{v}_{0}{\gamma\:}_{0}$, where $\:{\gamma\:}_{0}$ denote the effective coefficient of friction per unit volume of the NLCE, and let $\:{v}_{0}$ denote the volume of a single NLCE strip. The mechanical friction is neglected here (Supplemental Fig. S5). The external load torque $\:{T}_{\mathrm{l}\mathrm{o}\mathrm{a}\mathrm{d}}$ is considered to estimate the available power output. Then, a stationary angular velocity, $\:{\dot{\psi\:}}_{s}=(T-{T}_{\mathrm{l}\mathrm{o}\mathrm{a}\mathrm{d}})/\gamma\:$, where the maximum angular velocity $\:T/\gamma\:$ is realized when $\:{T}_{\mathrm{l}\mathrm{o}\mathrm{a}\mathrm{d}}=0$ and $\:\dot{\psi\:}=0$ when $\:{T}_{\mathrm{l}\mathrm{o}\mathrm{a}\mathrm{d}}=T$. Because $\:{\dot{\psi\:}}_{s}$ and $\:T$ can be experimentally determined, $\:\gamma\:$ can also be estimated and discussed. For further understanding the engine drive mechanism in terms of the torque balance, refer to the explanation in Supplemental Fig. S4. As described in Supplemental Note 2, the total energy efficiency is also derived with other related powers as $\:{\eta\:}_{\mathrm{t}\mathrm{o}\mathrm{t}}={P}_{\mathrm{l}\mathrm{o}\mathrm{a}\mathrm{d}}/{P}_{\mathrm{i}\mathrm{n}}$, with which related values can be estimated using the experimentally obtained values.

### Engine operation

The typical rotations of our prototype NLCE rotary engines are shown in Supplemental Movie 1. The dynamics were investigated by changing some parameters here. Firstly, the conditions of the initially applied strain are explained. For most of experiments, the condition called T1, in which $\:({e}_{\mathrm{m}\mathrm{i}\mathrm{n}},{e}_{\mathrm{m}\mathrm{a}\mathrm{x}})=(0.70,\:1.04)$ through the rotation of 2π, was used, where $\:({e}_{\mathrm{m}\mathrm{i}\mathrm{n}},{e}_{\mathrm{m}\mathrm{a}\mathrm{x}})$ are minimum and maximum strains at $\:\psi\:=0$ and π, respectively, through a revolution (Fig. 4c). The higher strain condition called T2, in which $\:({e}_{\mathrm{m}\mathrm{i}\mathrm{n}},{e}_{\mathrm{m}\mathrm{a}\mathrm{x}})=(0.99,\:1.91)$, was adopted for *N* = 4 (referred to as N4), to examine the effect of the initially applied strain, or pre-tension. Since the engine radius is constant, the change in NLCE length during rotation is identical at $\:\varDelta\:L=5.86\:\mathrm{m}\mathrm{m}$. However, because the initial elongation at installation differs, the resulting change in strain, $\:{e}_{\mathrm{m}\mathrm{a}\mathrm{x}}-{e}_{\mathrm{m}\mathrm{i}\mathrm{n}}$, is greater for T2. The corresponding variation is illustrated by arrows on the force-extension curve in Fig. 2a.

**Fig. 5:**
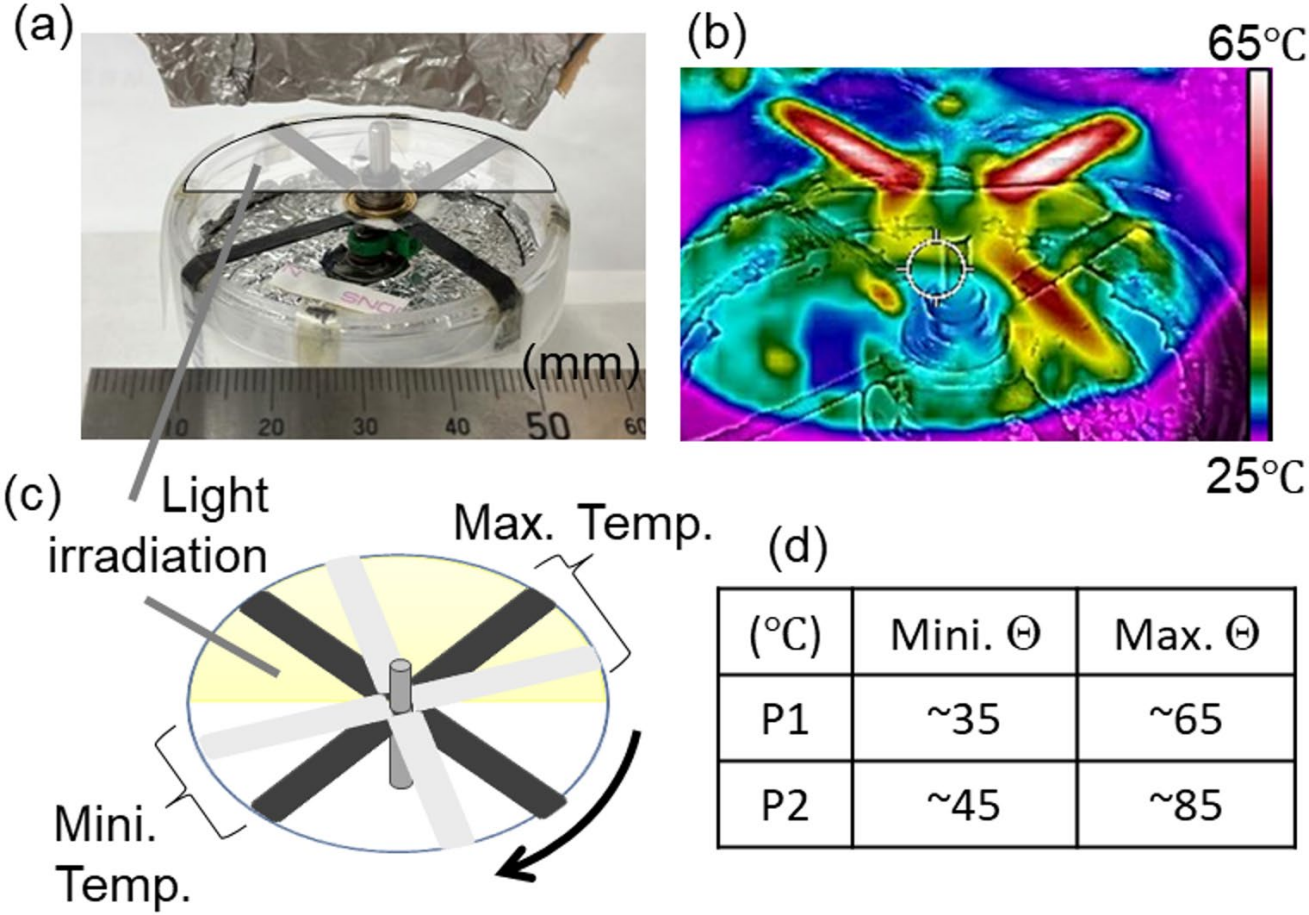
Light-powered 4-stroke rotary NLCE engine. (**a**) The engine overview (N4) on operation. (**b**) Temperature distribution (thermometric image) of N4 engine with the light power of P1 and (c) schematic illustration of the temperature. (**d**) Typical minimum and maximum temperatures with the light power of P1 (306 mW/cm^2^) and P2 condition (610 mW/cm^2^).

In Fig. 5, an example of the present NLCE rotary engine system (N4) under operation is shown with the temperature image (Fig. 5b). The regions showing maximum and minimum temperatures *Θ* are schematically shown in the illustration (Fig. 5c) and the typical minimum and maximum *Θ* s with the light powers of P1 (306 mW/cm^2^) and P2 condition (610 mW/cm^2^) at a room temperature of 25 °C are also shown in Fig. 5d. Under both light irradiation conditions, P1 and P2, the maximum temperature exceeded $\it \:{\varTheta\:}_{c}$. Even during the air-cooling interval while rotating, the temperature did not cool down to room temperature but remained below $\it \:{\varTheta\:}_{c}$.

**Fig. 6:**
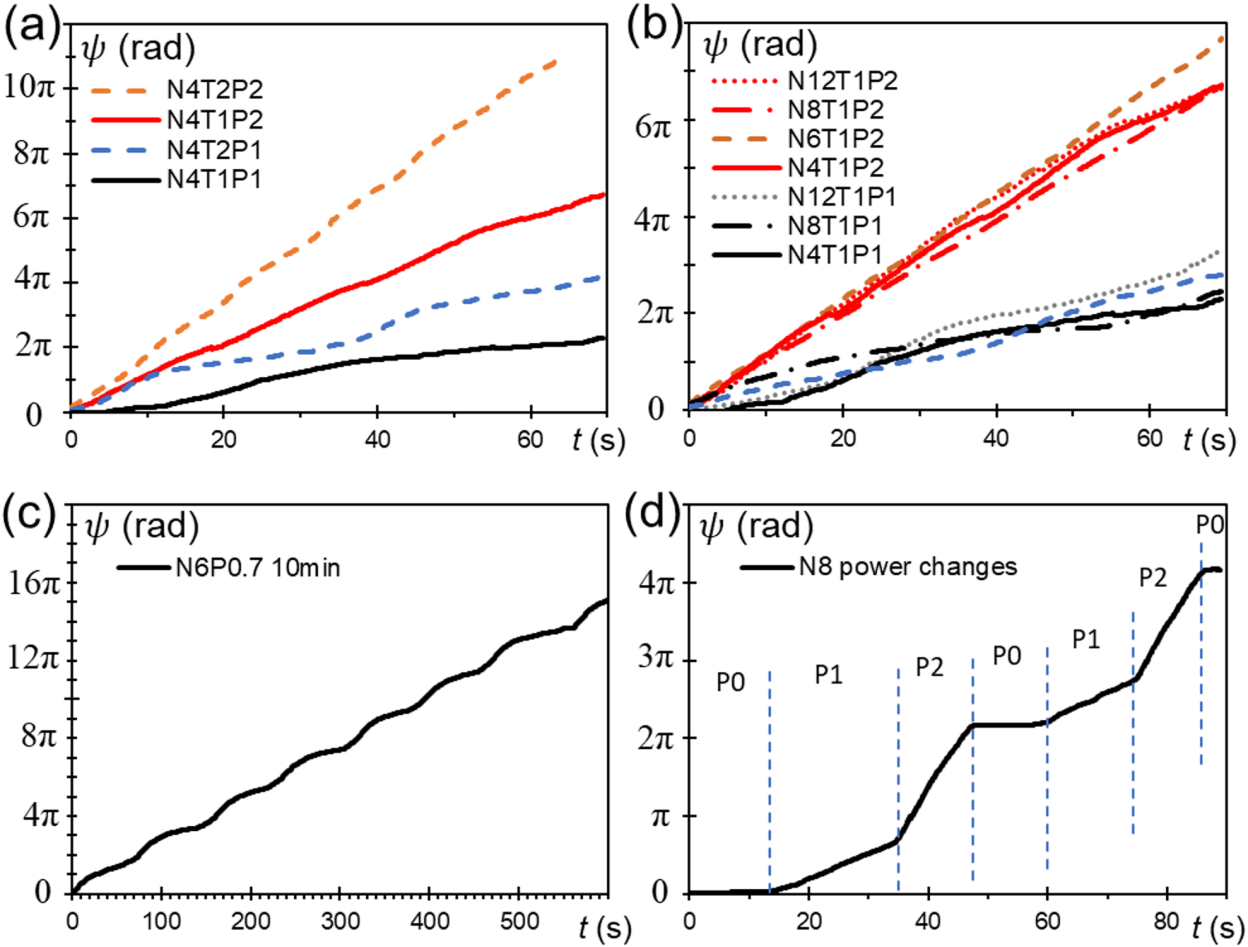
Time-dependent rotational angles *ψ*(*t*). (**a**) Data with different initially applied strain, T1 and T2, and light powers, P1 and P2 for N4. (**b**) Data with P1 and P2 for *N* = 4, 6, 8, and 12, (N4, N6, N8 and N12). (**c**) Data on N6, T1 and P0.7 (slightly lower light power) for 10 min. (**d**) Data on N8 and T1 changing the light power.

In Fig. 6, the time-dependent rotational angles $\:\psi\:\left(t\right)$ for various conditions, in which the different initially applied strain, T1 and T2 for N4, *N =* 4, 6, 8, and 12, (N4, N6, N8 and N12) and light powers of P1 and P2, are shown. The longer time data (for 10 min) for N6 with slightly lower light power (P0.7) shown in Fig. 6c (see also Supplemental Movie 2) suggests the stable operation of the NLCE engine. Although the dynamics over even longer periods will be modulated by the degree of long-term stress relaxation in the NLCE^30,31^, it has not been tested such extended periods here (see Supplemental Fig. S6 for the expected long-term engine fatigue). When changing the light power, the data (Fig. 6d) shows quick responsiveness where the rotational speed changes within seconds to follow the input power change (see also Supplemental Movie 3). This indicates that characteristic timescale of the system $\:I/\gamma\:\sim1\:\mathrm{s}$ (see also Supplemental Note 2). This responsiveness, $\:I/\gamma\:$, would be modulated by changing inertia (e.g., by adding mass to the wheel working as flywheel) and viscoelastic properties of NLCE (e.g. using the main-chain as opposed to side-chain NLCE).

As expected from the theoretical model (Supplemental Note 2), the data roughly shows the linear increase at the constant light power input with slight fluctuation, indicating the stationary rotation at the roughly constant angular speed $\:\dot{\psi\:}$ read from the slope of the data. As can be clearly seen in Fig. 6c, the cycle of this slight fluctuation is often 2π, occurring once per rotation. This fluctuation is assumed to be caused by the imbalance in the strain of the NLCE, as mentioned at the end of Supplemental Note 2. If it were caused by the small number of *N*, it should appear at a cycle of 2π/*N*. It is believed that this fluctuation can be reduced by more precisely adjusting the applied strain of each NLCE.

**Fig. 7:**
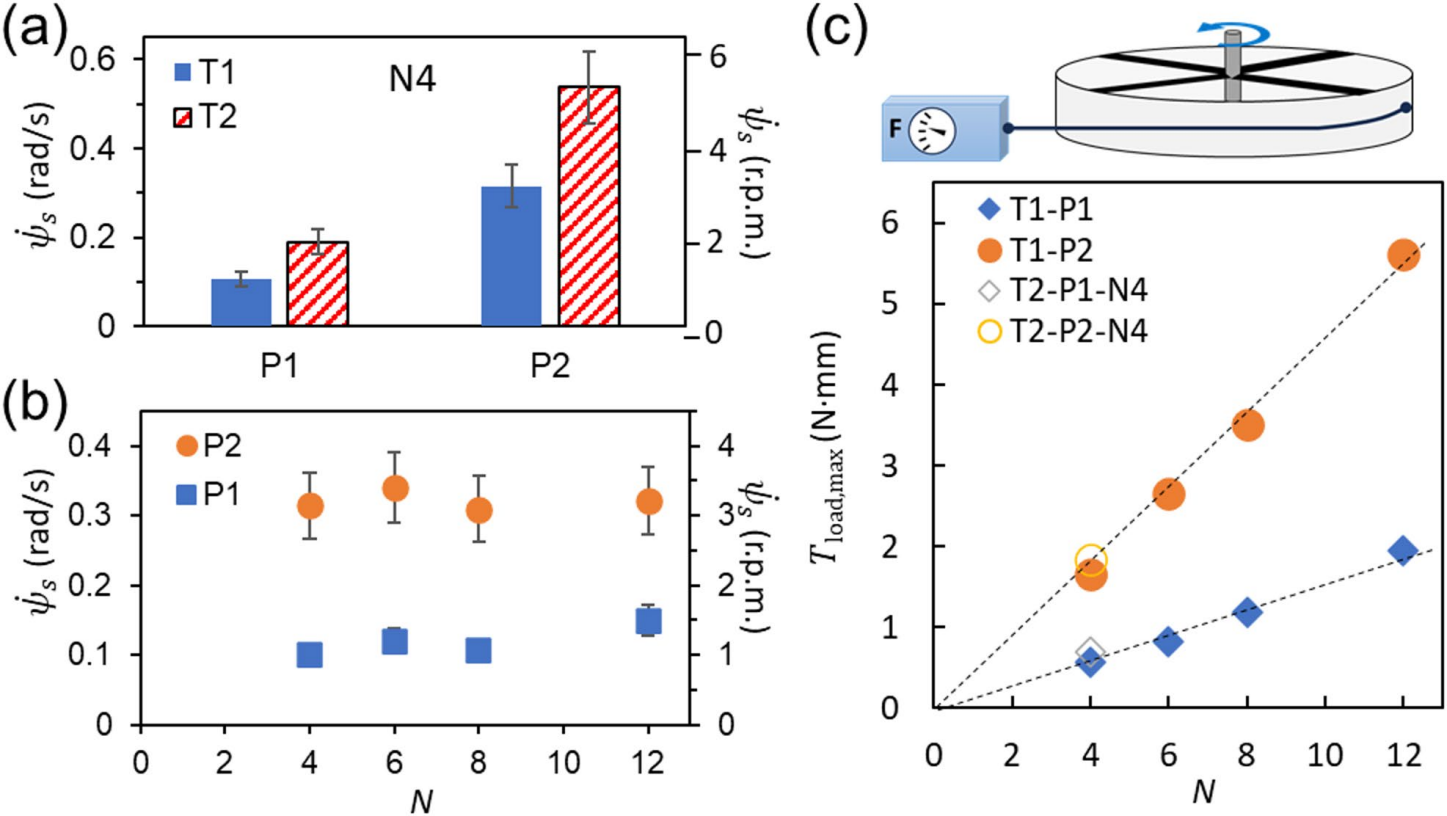
Steady rotation speed $\:{\dot{\boldsymbol{\psi\:}}}_{\boldsymbol{s}}$ and generated torque ***T***. (**a**) $\:{\dot{\psi\:}}_{s}$ with T1 and T2, and P1 and P2 for N4. (**b**) $\:{\dot{\psi\:}}_{s}$ with P1 and P2 for N4, N6, N8 and N12. (**c**) Maximum load (the blocking torque) at $\:{\dot{\psi\:}}_{s}=0,$ $\:{T}_{\mathrm{l}\mathrm{o}\mathrm{a}\mathrm{d},\mathrm{m}\mathrm{a}\mathrm{x}}=T$ on with P1 and P2 for N4, N6, N8 and N12. The method for measuring $\:{T}_{\mathrm{l}\mathrm{o}\mathrm{a}\mathrm{d},\mathrm{m}\mathrm{a}\mathrm{x}}$ is shown in the schematic illustration at the top. The force was measured when the rotation stops. (r.p.m.: rotation per minute)

The values of steady rotation speed $\:{\dot{\psi\:}}_{s}$ without load, which are obtained from $\:\psi\:\left(t\right)$ data shown in Fig. 6a, b, are summarized in Fig. 7a, b. In Fig. 7c, the generated net torque *T*, which is characterized as $\:{T}_{\mathrm{l}\mathrm{o}\mathrm{a}\mathrm{d},\mathrm{m}\mathrm{a}\mathrm{x}}$, torque at $\:{\dot{\psi\:}}_{s}=0$, are plotted with respect to *N*.

First, the effect of light power input, P1 and P2, is mentioned. According to the theoretical results of $T \propto r\Delta fN$ and $\:{\dot{\psi\:}}_{s}=T/\gamma\:$ with $\dot{\psi }_{s} \propto r\Delta fN/\gamma$, $\:{\dot{\psi\:}}_{s}\propto\:r\Delta\:fN/\gamma\:$ (Supplemental Note 2). Here $\:\varDelta\:f$ increases with light power because the higher light power induces the larger temperature difference, leading to the higher $\:\Delta\:f$. Although the relationship between $\:\Delta\:f$ and light power would be nonlinear because the relation between the light power and temperature, and the temperature-dependence of the force-extension curve, are both non-linear^6^, the result is qualitatively consistent with what is expected by the theoretical model; the greater the light power, the greater the generated torque and the faster the rotation. The light power corresponds to the throttle of this engine operation, as shown in Fig. 6d and Supplemental Movie 3.

Due to technical limitations, we were unable to adjust the optical power in fine increments, so detailed data on the minimum optical power required for driving was not obtained. However, it is estimated to be approximately one-third of that of P1, around 100 mW/cm^2^, which is comparable to that of sunlight, and thus, possibly with a lens, even sunlight may easily drive the present NLCE engine.

Next, the effect of the initially applied strain, T1 or T2, upon installing the NLCE is mentioned. As a technical issue, when the light power is high, such as at P2, the NLCE temperature can rise to 90 °C or higher, making fracture at the NLCE clamp section more likely for T2 case. Therefore, experiments increasing the applied strain beyond T2 were difficult with the current system. Consequently, this study investigated only the effects of two applied tensions, T1 and T2. The results showed that the blocking torque at $\:{\dot{\psi\:}}_{s}=0$, which corresponds to the net generated torque *T*, increased by approximately 10% at T2 compared to T1, so this change is minor. As discussed in the theoretical model section (Supplemental Note 1), $\:T\propto\:\varDelta\:f$, where $\:\varDelta\:f$ is the force difference in the force-extension curves between high and low temperatures (Fig. 2b). This result is consistent with the observation that the slopes of the force-extension curves at high and low temperatures are similar, *k*. In other words, the experimental results also indicate that $\:T$ is insensitive to the initially applied tension.

On the other hand, the zero-load steady rotation speed $\:{\dot{\psi\:}}_{s}$ increased by a factor of 1.7 from T1 to T2. Despite the minimal torque increase, this $\:{\dot{\psi\:}}_{s}$ rising by approximately 1.7 times suggests, according to the equation, $\:{\dot{\psi\:}}_{s}=T/\gamma\:$ with $\:{T}_{\mathrm{l}\mathrm{o}\mathrm{a}\mathrm{d}}=0$, that the effective friction coefficient $\:\gamma\:$ may have decreased. Fundamentally, this $\:\gamma\:$ is thought to be primarily caused by resistance to the rotational motion of the nematic director in the nematic phase temperature region, which is the lower temperature side. Therefore, the higher uniaxial strain state, where the nematic director rotates less, suggests a potential reduction in viscous resistance. Regarding applied strain and viscoelastic resistance, when considering optimization of NLCE engine performance, it is necessary to construct a system that suppresses rupture to obtain more systematic data, which remains a future research topic.

**Fig. 8:**
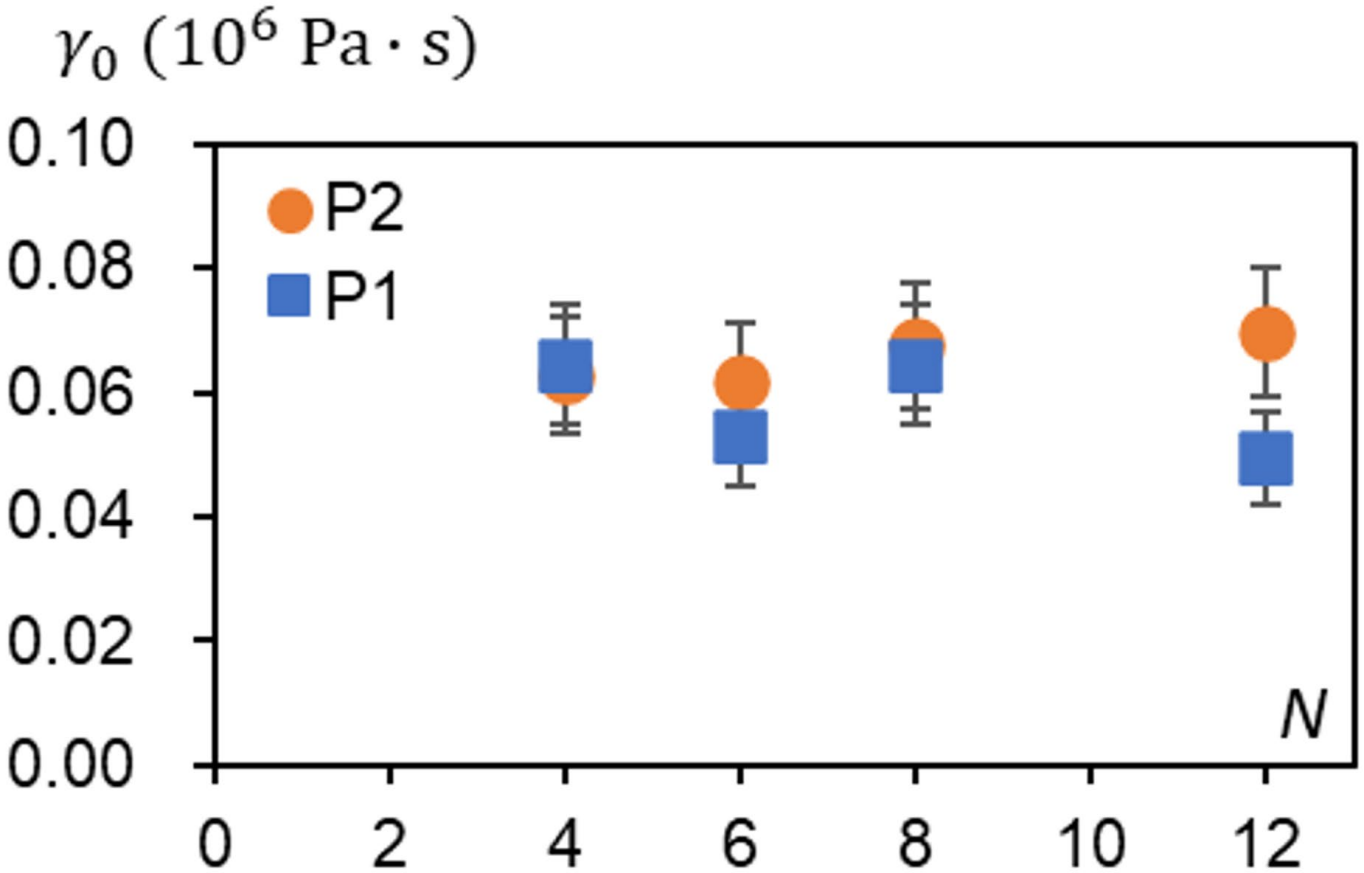
Estimated friction coefficient per unit volume of NLCE, $\:{\boldsymbol{\gamma\:}}_{0}$. The values lie roughly around 0.06 MPa.s.

Next, we discuss the results shown in Fig. 7b, where $\:{\dot{\psi\:}}_{s}$ remains unchanged even as *N* increases, along with the generated net torque results shown in Fig. 7c. According to the theoretical results of $\dot{\psi }_{s} = T/\gamma \sim r\Delta fN/\pi \gamma \propto r\Delta fN/\gamma$, where $\:r$ and $\:\varDelta\:f$ are constants for a fixed light power, the present result supports $\:\gamma\:\propto\:N$ as expected in the theoretical section as $\:\gamma\:=N{v}_{0}{\gamma\:}_{0}$, with which the effect of *N* is cancelled. Thus, $\dot{\psi }_{s} = T/\gamma \sim r\Delta f/\pi v_{0} \gamma _{0} \propto r\Delta f/v_{0} \gamma _{0}$. From the experimental data ($\:T,N,{v}_{0},{\dot{\psi\:}}_{s}$), the internal friction per unit NLCE volume, $\:{\gamma\:}_{0}$, can be estimated using the relation, $\gamma _{0} \sim T/Nv_{0} \dot{\psi }_{s}$, where $\:{v}_{0}\sim21\times\:{10}^{-9}\:{\mathrm{m}}^{3}$. The estimated $\:{\gamma\:}_{0}$ for P1 and P2 cases shown in Fig. 8 suggests that much of the data lies roughly around $\:0.06\:\mathrm{M}\mathrm{P}\mathrm{a}\cdot\:\mathrm{s}$. This further implies that $\:{\gamma\:}_{0}$ is one of the unique effective viscosity indices for this particular NLCE engine system and is directly related to the viscoelastic properties of the NLCE used. It is worth noting that this value is of the similar order of magnitude as the loss modulus (~ 0.12 MPa) at around 30 °C characterized with DMA, divided by the frequency of 1 Hz (1/s) (Fig. 1d-e).

Regarding the present angular or rotational speed $\:{\dot{\psi\:}}_{s}$, due to the enhanced viscoelasticity of this NLCE, values of around several r.p.m. have been observed, which are slower than those typically seen with conventional rubber^20^ with those up to 10–20 r.p.m. This study has revealed that this is unavoidable given the characteristics of the nematic phase in NLCE. On the other hand, as shown in Fig. 7c, high torque can be generated. Therefore, even at low rotational speeds, it is possible to produce high-speed motion using gears or similar mechanisms. With appropriate design considerations, this system could potentially achieve higher performance than engines using conventional rubber.

**Fig. 9:**
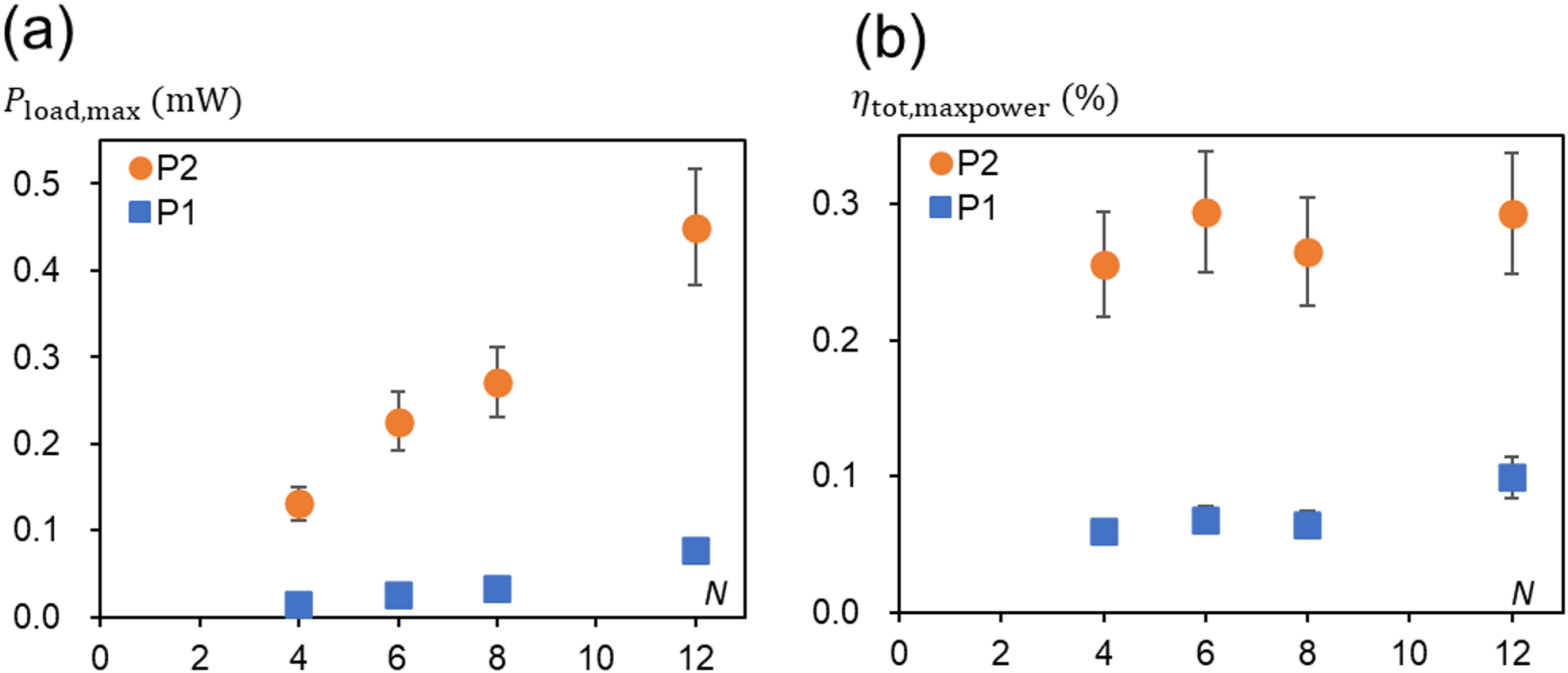
Estimated maximum available power output and overall energy efficiency. (**a**) $\:{P}_{\mathrm{l}\mathrm{o}\mathrm{a}\mathrm{d},\mathrm{m}\mathrm{a}\mathrm{x}}={T}^{2}/4\gamma\:=T{\dot{\psi\:}}_{s}/4$. This clearly depends on *N* and light power input. (**b**) $\:{\eta\:}_{\mathrm{t}\mathrm{o}\mathrm{t},\mathrm{m}\mathrm{a}\mathrm{x}\mathrm{p}\mathrm{o}\mathrm{w}\mathrm{e}\mathrm{r}}={P}_{\mathrm{l}\mathrm{o}\mathrm{a}\mathrm{d},\mathrm{m}\mathrm{a}\mathrm{x}}/{P}_{\mathrm{i}\mathrm{n}}$.

Then considering the present rotary engine for practical rather than educational purposes, it is important to evaluate the work output per unit time, that is power. To obtain useful work, the engine must run against a certain load, the power output returning back to zero at a “blocking load” when no rotation is taking place. The theoretical model (Supplemental Note 3) suggests that the maximum power output is obtained at $\:{T}_{\mathrm{l}\mathrm{o}\mathrm{a}\mathrm{d}}^{*}=T/2$ as $\:{P}_{\mathrm{l}\mathrm{o}\mathrm{a}\mathrm{d},\mathrm{m}\mathrm{a}\mathrm{x}}={T}^{2}/4\gamma\:=T{\dot{\psi\:}}_{s}/4$. Using the experimentally obtained values of *T* and $\:{\dot{\psi\:}}_{s}$, $\:{P}_{\mathrm{l}\mathrm{o}\mathrm{a}\mathrm{d},\mathrm{m}\mathrm{a}\mathrm{x}}$ is calculated and shown in Fig. 9a. As expected, the available power can increase almost linearly with *N* and input light power. The mechanical properties of the NLCE that directly reflect this power are $\:\varDelta\:f$, the source of torque generation, and the friction constant $\:{\gamma\:}_{0}$. Therefore, when optimizing available power based on the properties of NLCE, the key point in its design will be to increase $\:\varDelta\:f$ and reduce $\:{\gamma\:}_{0}$ for a given temperature difference. Conversely, evaluating this power characteristic enables assessment of the NLCE’s performance as a continuously driven soft actuator material, demonstrating the potential for this pilot system to serve as such a platform.

The energy efficiency limited to the mechanical system $\:\eta\:$ at maximum power output, $\:{\eta\:}_{\mathrm{m}\mathrm{a}\mathrm{x}.\mathrm{p}\mathrm{o}\mathrm{w}\mathrm{e}\mathrm{r}}=50\%$ as derived in Supplemental Note 3, which is independent of system parameters. Therefore, it can be seen that an equivalent amount of power is lost as viscoelastic dissipation. Consequently, it can also be understood that, ideally, reducing the effective viscosity, friction of the system $\:\gamma\:$, directly leads to an increase in the power itself.

When considering overall energy efficiency $\:{\eta\:}_{\mathrm{t}\mathrm{o}\mathrm{t}}$, the incident light should be regarded as the original energy input, and in addition to the losses $\:{P}_{\mathrm{d}\mathrm{i}\mathrm{s}}$ due to viscoelasticity mentioned above, the losses incurred when the light energy heats the NLCE, $\:{P}_{\mathrm{l}\mathrm{o}\mathrm{s}\mathrm{s}}$, should also be considered. The total power supply $\:{P}_{\mathrm{i}\mathrm{n}}$ can be directly estimated from the light power and the light-receiving area of NLCEs, *a*_0_*N*/2 ~ $\:21N\times\:{10}^{-9}$ m^2^ ~ $\:21N\times\:{10}^{-5}$ cm^2^. The light powers used here are *P*_1_ = 306 mW/cm^2^ and *P*_1_ = 610 mW/cm^2^. Then, $\:\left({P}_{\mathrm{i}\mathrm{n}-\mathrm{P}1},{P}_{\mathrm{i}\mathrm{n}-\mathrm{P}2}\right)\:\sim\:\left(6.43N,12.81N\right)\:\left[\mathrm{W}\right]$. Then, the overall energy efficiency at maximum available power, $\:{\eta\:}_{\mathrm{t}\mathrm{o}\mathrm{t},\mathrm{m}\mathrm{a}\mathrm{x}\mathrm{p}\mathrm{o}\mathrm{w}\mathrm{e}\mathrm{r}}={P}_{\mathrm{l}\mathrm{o}\mathrm{a}\mathrm{d},\mathrm{m}\mathrm{a}\mathrm{x}}/{P}_{\mathrm{i}\mathrm{n}}={T}^{2}/4\gamma\:{P}_{\mathrm{in}}$, can be estimated as shown in Fig. 9b. The values of $\:{\eta\:}_{\mathrm{t}\mathrm{o}\mathrm{t},\mathrm{m}\mathrm{a}\mathrm{x}\mathrm{p}\mathrm{o}\mathrm{w}\mathrm{e}\mathrm{r}}$ for P1 and P2 cases are almost independent of *N* and the averages are ~ 0.072% and ~ 0.277%.

These are much smaller than the upper limit of this total energy efficiency is the Carnot efficiency, $\:{\eta\:}_{\mathrm{c}}\sim0.083\sim8.3\%$, estimated in Supplemental Note 3. The results indicate that the energy conversion efficiency when light energy raises the temperature of the NLCE is rather poor. Presently, the NLCE is exposed to the external environment, making it prone to easy heat radiation, and no optimization has been made regarding light absorption either; hence, efficient heating is likely not being achieved. Furthermore, P1 exhibits an overall efficiency approximately one-quarter lower than P2. This is thought to be due to the slower rotation in P1, which reduces the light-to-heat exchange efficiency during continuous illumination at a constant intensity. To improve efficiency, efforts to reduce the energy loss associated with this temperature rise, as well as maintaining the high temperature state of NLCEs at the appropriate position and duration through appropriate thermal insulation will be necessary going forward.

Finally, comments on the relationship between engine scale and generated torque or output power would be meaningful for future engine design. Consider a case where all scales become $\:\lambda\:$ times larger (*N* is constant here). Since generated net torque is $\:T\propto\:r\varDelta\:fN$, $\:r$ becomes $\:\lambda\:r$, and $\:\varDelta\:f$ becomes $\:{\lambda\:}^{2}\varDelta\:f$ as the width and thickness of the NLCE increase proportionally. Thus, $\:T$ becomes $\:{\lambda\:}^{3}T$, meaning generated torque will increase significantly as the size increases. However, the internal friction also scales proportionally to the NLCE volume $\:{v}_{0}\propto\:{\lambda\:}^{3}$ as $\:\gamma\:=N{v}_{0}{\gamma\:}_{0}$, thus scaling as $\:{\lambda\:}^{3}$. Therefore, using the same NLCE, it can be expected that the angular velocity $\:{\dot{\psi\:}}_{s}=(T-{T}_{\mathrm{l}\mathrm{o}\mathrm{a}\mathrm{d}})/\gamma\:$ will be scale independent. Nevertheless, the available power $\:{P}_{\mathrm{l}\mathrm{o}\mathrm{a}\mathrm{d},\mathrm{m}\mathrm{a}\mathrm{x}}={T}^{2}/4\gamma\:$ scales as $\:{\lambda\:}^{3}$ while suppressing the friction term. Thus, increasing it with the increase of the input energy allows extraction of greater work.

The NLCE engine in this study has demonstrated steady-state operation at a small size, approximately 8 cm in diameter, fitting in the palm of our hand. However, with conventional rubbers, the effective force $\:\varDelta\:f$ at the temperature difference used here is much smaller compared to the present NLCE (see Supplemental Fig. S1), making operation at this size difficult. Indeed, Strong’s introduction^20^ cites examples with diameters about five times larger. This small rotary rubber engine could only be realized using NLCEs, representing a key feature that was clearly demonstrated for the first time through this theoretical modelling and experiments.

## Conclusions

In conclusion, the experimentally constructed light-driven rotary engine based on NLCE was analyzed alongside a simplified theoretical model. The focus was on how performance characteristics are utilizing unique properties of NLCE. The result confirmed steady rotational motion (a few cycles per minute) at a small scale unattainable with conventional rubbers. However, the rotational speed was approximately one order of magnitude lower than that of a standard rubber engine. This was attributed to the high internal friction exhibited by NLCE during elongation at low temperatures. Nevertheless, the ability to achieve steady rotation at this low speed is a significant result. This is because, even at low rotational speeds, torque and power output could be increased by varying the number of NLCE elements, light power, or the engine size. This performance capability is crucial when performing external work against the load. Furthermore, if desired, rotational speed can be modified via gear mechanisms, meaning low rotational speeds are not fatally detrimental to the engine performance. However, energy efficiency was found to suffer significant losses during the process of converting light into a temperature difference, presenting a future engineering challenge. The theoretical framework presented here, spanning from force generation by individual NLCE elements to the energy efficiency of the engine system will prove useful for future NLCE engine design. Furthermore, as the mechanical properties of NLCE, including its viscoelasticity, can be evaluated through the performance assessment of this NLCE engine, the experimental setup and theoretical model can also serve as an evaluation platform for general NLCEs. This is particularly significant when optimizing the physical properties of NLCE for use as a soft actuator.

## Methods

###  Materials and preparation of NLCEs

 Previously reported methods^22,32,33^ were followed for preparing the NLCEs, which involved a Michael addition reaction between acrylates and thiols. The diacrylate monomer, 2-methyl-1,4-phenylene bis(4-(3-(acryloyloxy)propoxy)benzoate) (RM257), was purchased from Wilshire Technologies (Fig. 1a). Two thiol monomers, 2,2’-(ethylenedioxy) diethanethiol (EDDET) and pentaerythritol tetrakis (3-mercaptopropionate) (PETMP), were purchased from Sigma Aldrich. Triethylamine (TEA, Sigma Aldrich) was used as the Michael addition catalyst. Butylated hydroxytoluene (BHT, Sigma-Aldrich) was used as the radical scavenger to suppress unwanted radical polymerization reactions between acrylates. All chemicals were used in the as-received conditions without purification. RM257, EDDET, and PETMP were weighed at specific molar ratios of 50:48:1. BHT (0.5 wt%) were added. After the mixture was gently mixed at an elevated temperature (~ 80 °C) for ~ 10 min, TEA (1.5 wt%) was added to initiate the Michael addition reaction between the thiol and acrylate groups. The mixture was molded between two glass slides with 0.5-mm-thick spacers at 80 °C (isotropic phase) for 48 h. Notably, no surface alignment was required. The sample was subsequently peeled and annealed at 80 °C in a vacuum oven for 12 h. The sample was cooled thereafter to room temperature to obtain pre-crosslinked samples exhibiting polydomain patterns. Samples were cut and subsequently used to generate stripe domains. The surface was painted twice repeatedly with a black oil-based pen to impart photothermal conversion properties, dried thoroughly, and used for basic property analysis and engine fabrication with light irradiation.

Natural rubber strips with the thickness of 0.5 mm (GS-01, WAKI Sangyo) were used as reference material for several evaluations.

#### Differential scanning calorimetry (DSC)

Non-painted NLCE samples (~ 15 mg) were loaded into standard aluminum DSC pans for DSC analysis (DSC8500, PerkinElmer). The sample was subjected to a heating and cooling cycle between − 60 and 100 °C at a rate of 5 °C/min.

#### Dynamic mechanical analysis (DMA)

The dynamic mechanical tests were performed on a DMA1 (Mettler Toredo), in the shear sandwich and the tension modes, with samples of 0.5 mm film thickness. For the shear mode, a circular sample (diameter of 10 mm) was used. The simple shear strain of 1% was applied at frequencies of 1, 10, and 100 Hz. Data was acquired on cooling at the rate of at − 5 °C/min from 100 to typically − 20 °C.

#### Force-extension (Stress–strain) response

The stress–strain curves of the NLCE films in the tensile mode at room temperature were obtained using a commercial instrument (EMX1000, IMADA) at room temperature. The typical sample width and effective length were 5 mm and 10 ~ 20 mm, respectively. We primarily used the engineering strain $\:e=\left(L-{L}_{0}\right)/{L}_{0}$, where $\:L$ and $\:{L}_{0}$ are the effective and initial lengths of the sample strip, respectively, and engineering stress $\:\sigma\:$, which is the force divided by the initial cross-sectional area $\:{a}_{0}$. $\:{L}_{0}$ and $\:{a}_{0}$ refer to the values without external stress or strain at a nematic temperature of 22 °C.

####  NLCE rotary engine preparation

 The basic design of the present star-shaped type rotating engine follows the Wiegand rubber engine that is one of engines reviewed by Strong^20^. A prototype was handmade here. The crankshaft was fabricated by joining two stainless steel round bars with a diameter of 2.9 mm. The wheel was machined from a plastic Petri dish measuring 55 mm in diameter and 10 mm in height. The main shaft was mounted at the wheel’s center via a ball bearing, with the NLCE-clamped eyelet fixture ring mounted onto the crankshaft positioned on the ball bearing. The NLCEs were fixed to the eyelet fixture as strips 0.5 mm thick and 5 mm wide, with the other ends fixed at a predetermined length to the wheel’s edge at equal intervals around the circumference. Due to the clamping arrangement, the number of NLCEs, *N* = 12, represented the technical upper limit.

#### NLCE engine operation

White LED light (LED A4, Wuben, China) was shone onto half of the engine from above, causing rotational motion. The light intensity was measured using a power meter (Model 3664, HIOKI) at a wavelength of 530 nm. The temperature of the sample surface was monitored using an infrared camera (C2, FLIR). The rotation was analyzed by tracing a specific point on the edge of the wheel in a video using motion-capture PTV software (Degimo, Japan).

## Supplementary Information

Below is the link to the electronic supplementary material.

Supplementary Material 1

Supplementary Material 2

Supplementary Material 3

Supplementary Material 4

## Data Availability

The data that support the findings of this study are available from the corresponding author upon reasonable request.
