# Supplementary material for: A nematic liquid crystal elastomer rotary engine
Source: Sci Rep. 2025 Dec 23;16:3322. doi: 10.1038/s41598-025-33311-0 (PMC12835278; doi:10.1038/s41598-025-33311-0)
Supplement: Supplementary file 4 — Supplementary Material 4 [file 41598_2025_33311_MOESM4_ESM.pdf]

Supplemental Materials

A nematic liquid crystal elastomer rotary engine

Takuya Ohzono[1,2]*, *Hirohmi Watanabe*[3], *Eugene M. Terentjev*[4]

[1] Research Institute for Electronics and Photonics, National Institute of Advanced Industrial Science and Technology (AIST) 1-1-1 Higashi, Tsukuba 305-8565, Japan
[2] Clare Hall, University of Cambridge, Herschel Rd, Cambridge CB3 9AL, United Kingdom
[3] Research Institute for Sustainable Chemistry, AIST, 3-11-32 Kagami-yama, Higashi-Hiroshima, Hiroshima739-0046, Japan
[4] Cavendish Laboratory, University of Cambridge, J.J. Thomson Avenue, Cambridge, CB3 0HE, United Kingdom
E-mail: ohzono-takuya@aist.go.jp

Fig. S1. Reconstructed temperature-dependent force-extension curves of natural rubber.

Supplemental Note 1. Theoretical model on torque generation.

Fig. S2. Derivation of approximation, $T(r, \psi) \approx rf \sin\psi$.

Fig. S3. Torque generation by NLCEs.

Fig. S4. Schematic diagram to aid understanding of the torque balance under continuous clockwise rotation.

Supplemental Note 2. Theoretical model on stationary rotation dynamics.

Supplemental Note 3. Theoretical model on energy efficiency.

Fig. S5. Effects of mechanical parts, ball bearings, other than NLCE on rotational friction.

Fig. S6. Long-term light-induced force decay and expected engine fatigue.

Supplemental Movie 1. Typical NLCE engine rotation.

Supplemental Movie 2. NLCE engine rotation upon light power change.

Supplemental Movie 3. NLCE engine rotation upon light power change.

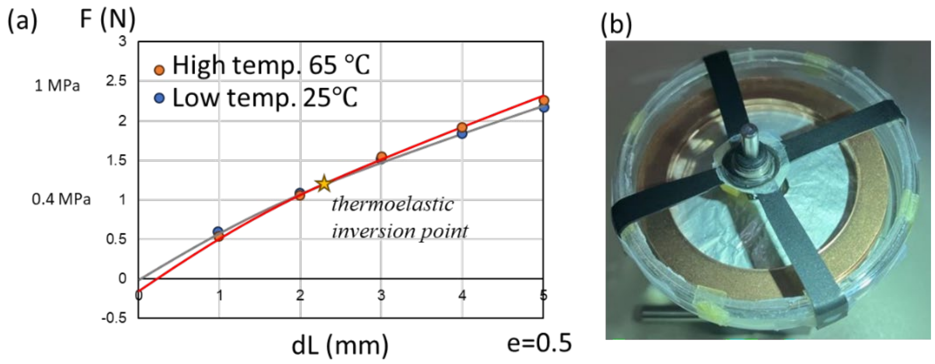

Fig. S1. Reconstructed temperature-dependent force-extension curves of natural rubber. (a) Experimental results with the natural rubber strip with the initial thickness of 0.5 mm, initial length of 10 mm, and width of 5 mm. At fixed extensions (dL = 1, 2, 3, 4, and 5 mm), the light-induced stress changes were measured. Upon light irradiation, the temperature increased from 25 to ~65°C. From the data, the temperature-dependent force-extension curves were reconstructed (solid curves). There exists thermoelastic inversion point at dL~2.2 mm, over which the stress increases upon temperature rise. At e = 0.5 (dL = 5 mm), the stress increases approximately +5% (~0.1 N). (b) The natural rubber rotary engine with N = 4, which possesses exactly the same dimensions as the NLCE engines in this study. The minimum strain of 0.5 was chosen. However, it exhibited no movement whatsoever under all the conditions in which the NLCE engine in this study operated.

Supplemental Note 1. Theoretical model on torque generation.

Torque component from single NLCE strip. First, an overview is provided of the rotational torque fluctuations generated by the tension changes associated with the extension and contraction of a single NLCE strip during the process of forcibly rotating the wheel, assuming no temperature change ($\Theta = const.$). In this case, a single NLCE installed on the wheel ($N =$ 1) (Fig. 4c) will periodically change its length, $L(\psi)$ (Fig. 4d), as the wheel is forced to rotate

($\psi = 0 \sim 2\pi$), causing the change in tension force, F_c, according to $L(\psi)$. The relationship between force and extension can be determined from the strain-stress test of a single NLCE at a fixed temperature as shown in Fig. 2a. On the theoretical model, the relationship is simplified by assuming a linear spring with a spring constant k and an force offset $-\Delta f$ only at low Θ, as $F_c = k[L(\psi) - L_0] - \Delta f$ (low Θ), and $F_c = k[L(\psi) - L_0]$ (high Θ), where Δf is the force difference between high and low Θ at fixed length, L_0 is the natural length of the NLCE without constraint, and only the case of $F_c \geq 0$ is considered (Fig. 2b).

Given the tensile force on the NLCE, F_c, the tangential force at the wheel edge is calculated as $F_p = F_c \cos\phi$, where ϕ is the angle between the wheel tangential direction and long axis direction of the NLCE (Fig. 4c). Note that both F_c and ϕ are functions of the rotation angle ψ. Thus, the torque on the wheel is expressed as $T(r,\psi) = rF_p(\psi) = rF_c(\psi)\cos\phi(\psi)$. As the analytical expression of $T(r,\psi)$ is not available, it can be calculated numerically. Nevertheless, we find that $T(r,\psi)$ is well approximated as $T(r,\psi) \approx rf\sin\psi$, where $f = F_c\left[L\left(\frac{\pi}{2}\right), \Theta\right] d/\sqrt{d^2 + r^2}$ (see Supplemental Fig. S2) and is independent of ψ. Here, $F_c(\psi)(> 0)$ is maximized at $\psi = 0$ and minimized at $\psi = \pi$, and $\cos\phi(\psi) = 0$ at $\psi = 0, \pi$, and $\cos\phi(\psi)$ is maximized at $\psi = \pi/2$, then it can be found that the $\sin\psi$-type function is acceptable.

In the present case, the positive/negative value of $T(r,\psi)$ induces clockwise/counterclockwise rotation when it is free of rotational constraint. It is clear that in the case of the single NLCE ($N = 1$) the stable equilibrium point is $\psi = 0$ and the point $\psi = \pi$ is also equilibrium but unstable. Note that this equilibrium points are independent of temperature. Thus, the single NLCE case is not able to create stationary rotation assuming the small inertia, and at least multiple NLCEs are required for it. Note that if there were significant inertia, it would be feasible with an engine that functions like a pendulum, sustaining motion in the manner employed in pendulum clocks.

Net torque from multiple NLCE strips. The case with $N = 2$, where two NLCE strips are quipped at the counterpart position, is considered. The effective lengths of these NLCEs are the same here in their natural state, and there is no qualitative difference between the two. When the temperature Θ is constant, the net torque $T(\psi) = \sum_i^N T_i = rf_1 \sin\psi_1 + rf_2 \sin\psi_2 = rf(\sin\psi + \sin(\psi + \pi)) = 0$, where $\psi = \psi_1 = \psi_2 + \pi$ and $f_1 = f_2 = f = F_c\left[L\left(\frac{\pi}{2}\right), \Theta\right] d/\sqrt{d^2 + r^2}$. This indicates that the torque components are cancelled each other and any wheel positions are stable. Thus, in the case of an even number of multiple NLCEs $N = 2j$ (j: integer), the torque components of the opposing NLCEs are always cancelled and all wheel positions, any value of ψ, are stable.

Next, let us consider the general case with temperature gradient and $N = 2$, where one of the NLCEs are exposed to light and heated, thereby increasing their tension. Assuming that NLCEs are at two different temperatures, $\Theta_H(> \Theta_c)$ and $\Theta_L(< \Theta_c)$ depending on the presence or absence of light irradiation, NLCEs at Θ_H are in the states with higher tension. When the temperature differs between two opposing NLCEs, f for NLCEs in the above torque equation becomes different, $T \neq 0$ except for at ψ_i =0, π, and the wheel rotation is driven. Moreover, if $N \geq 4$ with Θ depending on the position, T no longer becomes zero at any wheel position and the wheel rotation becomes continuous under light irradiation at a fixed region. In other words, the broken balance on the torque equation and the maintenance of the unbalanced state are keys for continuous wheel rotation, and it is triggered by providing a temperature difference between NLCEs placed in opposing positions within this engine. (If there were a mechanism utilising inertia, such as a flywheel, rotational motion could be achieved even with $N = 2$.)

As examples, for the case with $N = 2$, net torque is expressed as $T(\psi) = rf_1 \sin\psi_1 + rf_2 \sin\psi_2 = r(f_1 - f_2)\sin\psi$, where $\psi = \psi_1 = \psi_2 + \pi$ and for the case with $N = 4$, $T(\psi) = rf_1 \sin\psi_1 + rf_2 \sin\psi_2 + rf_3 \sin\psi_3 + rf_4 \sin\psi_4 = r[(f_1 - f_3)\sin\psi + (f_2 - f_4)\sin(\psi - \pi/2)]$, where $\psi = \psi_1 = \psi_2 + \pi/2 = \psi_3 + 2\pi/2 = \psi_4 + 3\pi/2$. Here, to simplify the theoretical

model regarding the light-induced temperature distribution, we consider the case that temperature at the half part of the wheel with $\psi_i = 0{\sim}\pi$ is increased instantly to Θ_H and kept by partial light exposure (Fig. 4e) and other part is instantly cooled to Θ_L. This assumption is based on very fast response of NLCEs to the light regarding temperature increase and decrease. In real systems, temperature changes are not instantaneous, and the temperature distribution should have a gradient from Θ_L to Θ_H, suggesting that the present assumption is crude in terms of the temperature distribution. Nevertheless, the main states required for the engine operation are safely expressed here.

Then, $f_i = F_c\left[L\left(\frac{\pi}{2}\right), \Theta_i\right] d/\sqrt{d^2 + r^2} = f_H$ (for $\Theta_i = \Theta_H, \psi_i = 0{\sim}\pi$), or $= f_L$ (for $\Theta_i = \Theta_L, \psi_i = \pi{\sim}2\pi$), $f_H > f_L$ and $\Delta f = f_H - f_L$. Focusing on the torque component of a single NLCE $rf_i \sin\psi_i$, as the wheel rotates from $\psi_i = 0$ to 2π, in the first half $f_i = f_H$ and $\sin\psi_i > 0$ and in the second $f_i = f_L$ and $\sin\psi_i < 0$. Using this relationship to transform above net torque equation in the case of $N = 2$, $T(\psi) = r\Delta f|\sin\psi| \geq 0$ (Supplemental Fig. S3a) and of $N = 4$, $T(\psi) = r\Delta f(|\sin\psi| + |\cos\psi|) = r\Delta f(|\sin\psi| + |\sin(\psi - \pi/2)|) > 0$ (Supplemental Fig. S3b). These $T(\psi)$ are always positive and discontinuous with a period of π or π/2, respectively for $N = 2$ or 4, which drives a rotation regardless of the wheel position.

In accordance with the aforementioned approach, we proceed with a generalisation when N is even as $T(\psi, N) = \sum_i^N T_i = \sum_j^{N/2} T_j$, where $j = i/2$ and T_j is the sum of torque components from two opposing NLCE strips, $T_j = rf_j \sin\psi_j + rf_{j+N/2} \sin\psi_{j+N/2} = r\Delta f|\sin[\psi + (2\pi/N)(j-1)]|$ (Supplemental Fig. S3a, top). The technical point in this derivation is that by considering opposing NLCE pairs, which have the phase difference of π, as a single set (Supplemental Fig. S3a, top), T_j becomes the corresponding torque from a pair j. Shifting this by a phase difference of $2\pi/N$ and summing the results over j yields the net torque $T(\psi, N) = r\Delta f \sum_j^{N/2} |\sin[\psi + (2\pi/N)(j-1)]|$. This function oscillates depending on the wheel position

ψ with the periodicity of $2\pi/N$ as shown in Supplemental Fig. S3b. The average T_{av} over ψ, minimum, T_{min}, maximum, T_{max}, the difference, $T_{diff} = T_{max} - T_{min}$, and T_{diff}/T_{max} can be numerically calculated as a function of N and shown in Supplemental Fig. S3c-e.

These results reveal key theoretical information about the drive torque of this NLCE rotary engine. Firstly, the torque oscillation amplitude T_{diff} decreases with increasing N (Supplemental Fig. S3d-e). Secondly, the average torque T_{av} is approximately proportional to N and $r\Delta f$, that is, $T \sim r\Delta f N/\pi \propto r\Delta f N$. Furthermore, these two qualitative trends would be the same even when N is odd number. When torque oscillates, the rotation rate $\dot{\psi}$ is less likely to be constant, indicating that a higher N value is preferable for steady speed rotation. It also suggests that N and $r\Delta f$ should be increased for rotation with the higher load. In the later experiment, r (the system size) will be fixed so that the effects of N and Δf can be examined in practice. Here, Δf is more practically varied via changing mainly the light power and optionally initially applied tensile strain of NLCE strips from their natural state at installation. In the model, the area receiving light from the NLCE is not apparently considered. However, in experiments, as N increases, overlaps of NLCE strips occur, particularly near the centre, and clamping becomes practically more difficult. Therefore, in experiments, data acquisition is limited to N = 12 as the upper limit.

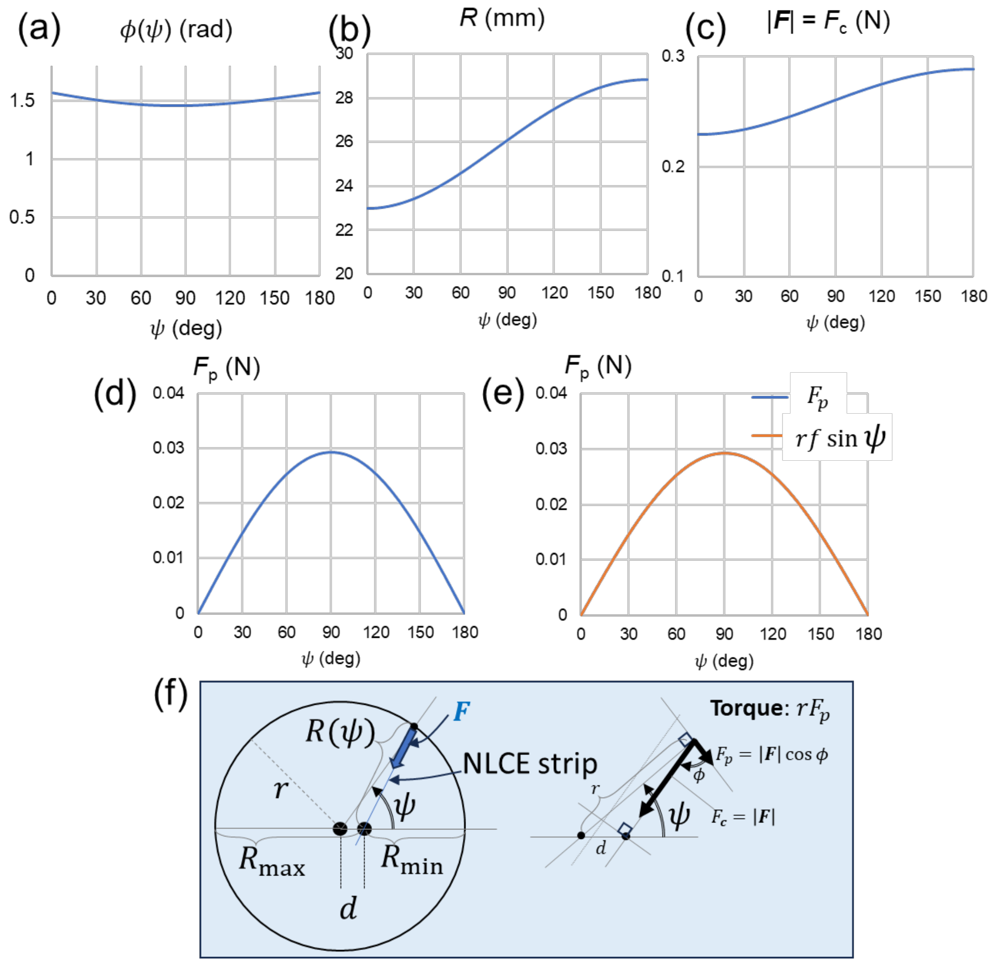

Fig. S2. Derivation of approximation, $T(r,\psi) \approx rf\sin\psi$. The torque on the wheel is expressed as $T(r,\psi) = rF_p(\psi) = rF_c(\psi)\cos\phi(\psi)$, where r is constant. (a) $\phi(\psi) = \tan^{-1}[(r - d\cos\psi)/d\sin\psi]$, and thus, $\phi(0) = \phi(\pi) = \pi/2$ and $\phi(\pi/2) \sim 0.928(\pi/2)$. Then, $\cos\phi(0) = \cos\phi(\pi) = 0$ and $\cos\phi(\pi/2) = d/\sqrt{d^2 + r^2} \sim 0.112$. (b) $R(\psi) = \sqrt{(r\sin\psi\,)^2 + (r\cos\psi - d)^2}$ and (c) $F_c(\psi) = k[L(\psi) - L_0]$, where $L(\psi) = R(\psi) - c$. Upon $\psi = 0\ \rightarrow \pi$, $R_{\min} \rightarrow R_{\max}$, and F_c increases similarly. (d) Finally, F_p appears as sine function. Since $F_p(\pi/2) \sim d/\sqrt{d^2 + r^2} \cdot F_c(\pi/2)$, the $F_p(\psi)$ can be approximated by a sine function, $rf\sin\psi$, where $f = F_p(\pi/2) \sim d/\sqrt{d^2 + r^2} \cdot F_c(\pi/2)$. (e) It can be confirmed that $F_p(\psi)$ produced by numerically calculated values and the one plotted by superimposing an approximate sine function $rf\sin\psi$ match so closely that they are indistinguishable. (f) The geometrical relation for convenience.

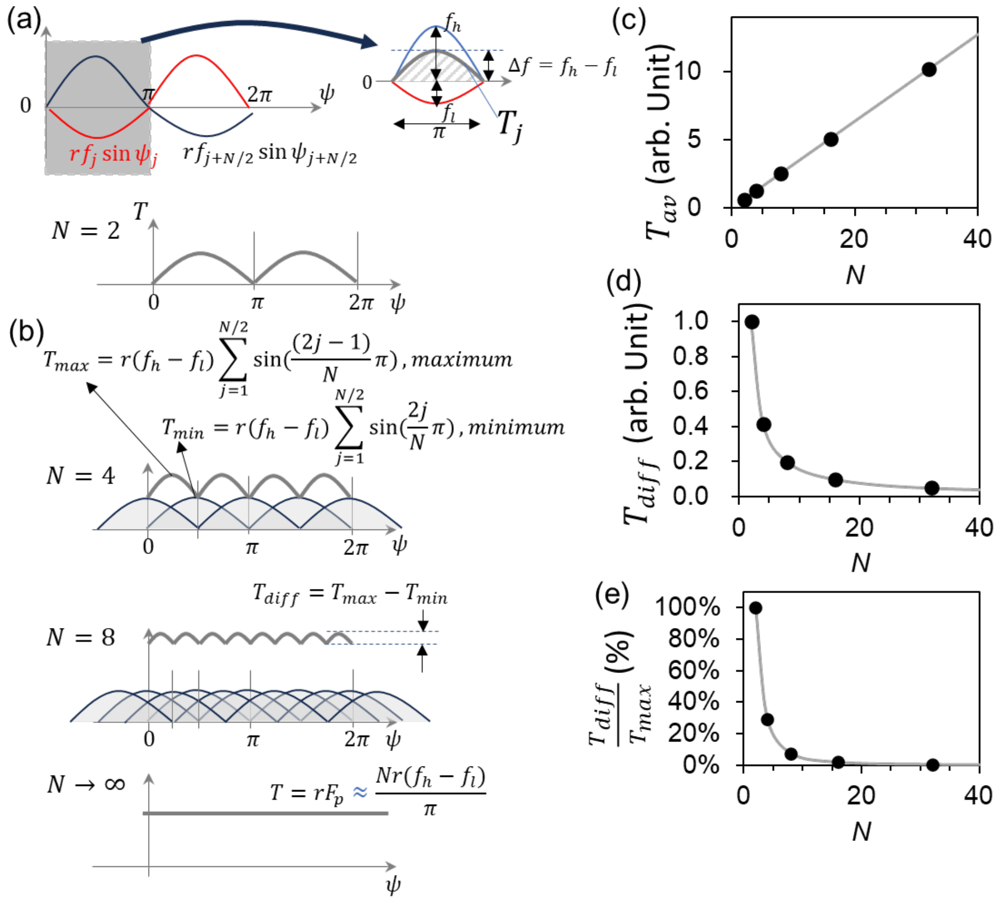

Fig. S3. Torque generation by NLCEs. Cases for (a) $N = 2$, (b) $N = 4$, 8, $\to \infty$. (c) Numerically calculated (c) $T_{av} \propto N$, (d) T_{diff} and (e) T_{diff}/T_{max} for different N.

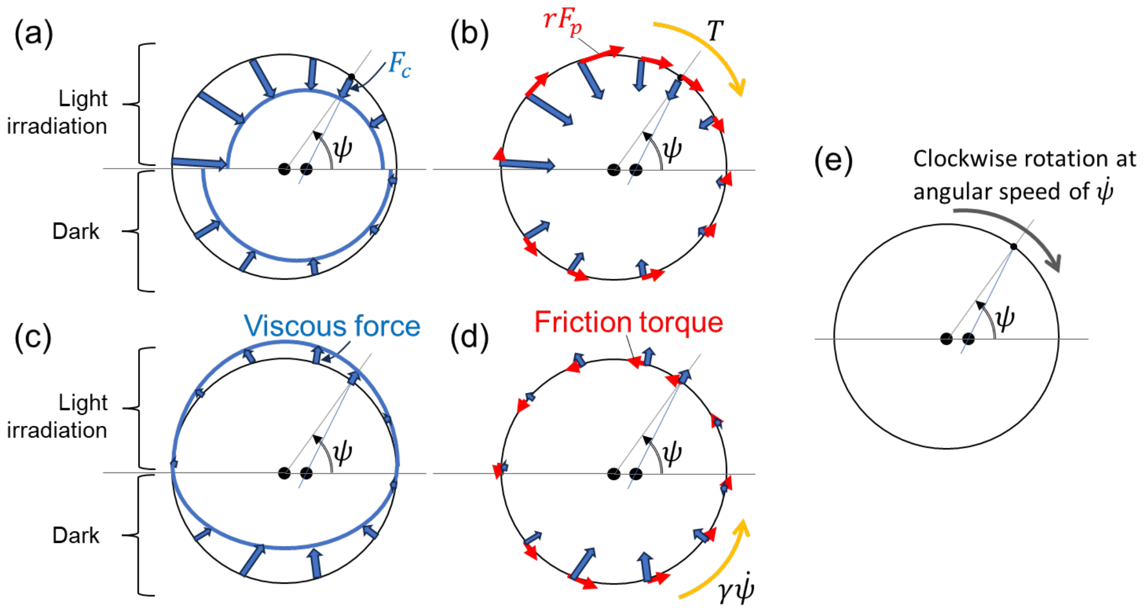

Fig. S4. Schematic diagram to aid understanding of the torque balance under continuous clockwise rotation. (a) ψ -dependent tensile force (F_c) along the NLCE strips (blue arrows). (b) ψ -dependent torque components $rF_p(\psi) = rF_c(\psi)\cos\phi(\psi)$ (red arrows) originating from tensile force along the NLCE strips shown in (a). The length is exaggerated for clarity. The light-illuminated side generates clockwise torque, while the dark side generates counterclockwise torque. Since the absolute value is greater on the light-illuminated side, the net torque produces the clockwise rotation. That is, the sum of this over all NLCEs along the perimeter equals to $T(r,\psi)$ (see also Fig. S3). (c) ψ -dependent viscous friction force (blue arrows) along the NLCE strips, which are not apparently considered for each NLCE but for the whole system as the frictional torque in the model as γ. Here, clockwise rotation is assumed, and resistance arises to counteract the length changes caused by this rotation. Note that the magnitude of this change is greatest at $\psi = \pi/2, 3\pi/2$, where the length change is large, and it becomes zero at $\psi = 0, \pi$ where there is no length change. Also note that in the dark area, the nematic phase results in a larger absolute value than in the illuminated area. (d) ψ -dependent torque components (red arrows) originating from viscous resistance force shown in (c). Sum of this over all NLCEs along the perimeter is $\gamma\dot{\psi}$. (e) Torque balance state $\gamma\dot{\psi} = T$ without

external load torque ($T_{\mathrm{load}} = 0$) under steady rotation (see also Fig. 4e). In the case with T_{load} under the overdamped dynamics, $\gamma\dot{\psi} = T - T_{\mathrm{load}}$, suggesting the decrease of rotation speed, that is, the decrease of friction torque.

Supplemental Note 2. Theoretical model on stationary rotation dynamics.

Dynamics and stationary rotation. As shown in Fig. 4e, the relationship between the temperature distribution applied to the NLCE rotary engine and the offset direction of the crankshaft generally determines the direction of rotation. Defining the temperature gradient vector from low to high temperatures and the offset vector from the true center towards the offset center of the crankshaft, the direction of rotation becomes that in which the temperature gradient vector rotates to lie along the offset vector, as shown in the figure, clockwise. That is, changing the light irradiation position to the lower side can induce reverse rotation.

Given the ψ -independent net torque $T \sim T_{av} (> 0)$ assuming larger number of N, the rotational dynamics can be easily discussed via the rotational equation of motion of the main wheel, $I\ddot{\psi} + \gamma\dot{\psi} = T - T_{\mathrm{load}}$, where the angular-velocity-dependent linear rotational friction of the system is apparently introduced. Note that from this point onwards, to align the experiment with the direction of rotation, we redefine the clockwise direction as the direction in which ψ increases. This is merely a difference in perspective, whether viewing the system from above or from below, and is not fundamentally relevant.

Because the viscoelasticity of NLCE is particularly large in the nematic phase, the effect is the main consideration as the source of friction. Other effects include rotational friction in mechanical parts other than the NLCE, e.g., ball bearings on shafts, but this can be assumed to be small (see Supplemental Fig. S5). Thus, γ may be the function of the effective volume of NLCEs. Let γ_0 denote the effective coefficient of friction per unit volume of the NLCE, and let

v_0 denote the volume of a single NLCE strip. Then, $\gamma = N v_0 \gamma_0$. The validity of this expression, along with experimental results, will be discussed later. Moreover, the external load torque T_{load} is also apparently included to consider the available mechanical work from the present NLCE engine.

The general solution of the rotational equation of motion with the initial condition $\dot{\psi}(0) = 0$ is, $\dot{\psi}(t) = [(T - T_{\mathrm{load}})/\gamma]\left(1 - e^{-\frac{\gamma}{I}t}\right)$, where I/γ is the characteristic timescale of the system. After sufficient time ($I/\gamma \ll t$), the corresponding overdamped dynamics ($I\ddot{\psi} \approx 0$) is realized showing a stationary angular velocity, $\dot{\psi}_s = (T - T_{\mathrm{load}})/\gamma$ (see also Supplemental Fig. S4). This suggests that the stationary rotation speed may be proportional to T, which would be parameterized by changing light power and/or initial strain of equipped NLCEs. Moreover, the maximum angular velocity T/γ is realized when $T_{\mathrm{load}} = 0$ and $\dot{\psi}(t) = 0$ when $T_{\mathrm{load}} = T$. On the other hand, the maximum load torque is reached to the driving net torque as $T_{\mathrm{load,max}} = T$ with $\dot{\psi} = 0$.

Experimentally, T can be determined by increasing T_{load}, since T_{load} in the condition where rotation stops equals T. Then, the friction coefficient γ of the system can be further determined from the observed maximum angular velocity $\dot{\psi}_{s-free} = T/\gamma$ with $T_{\mathrm{load}} = 0$, as $\gamma = T/\dot{\psi}_s$. These experimentally obtained *N*-dependencies of T and $\dot{\psi}_s$ will allow us to discuss the main origin of the friction coefficient γ. If the viscoelasticity of the NLCE were the primary cause of system friction, γ would increase along with *N*, as expressed as $\gamma = N v_0 \gamma_0$. Therefore, changing *N* would likely have little effect on the steady-state rotational speed. This is indeed what experimentally observed, as will be discussed in the experimental results section.

It is worth mentioning the issue with treating torque T as a constant here. In reality, T should depend on rotational speed $\dot{\psi}$. Qualitatively, at high speeds, both temperature rise and cooling take longer, resulting in a smaller Δf in each NLCE and thus a lower T. To explicitly incorporate this effect into the model, we need to introduce velocity dependence into T, as $\gamma\dot{\psi} =$

$T(\dot{\psi})$ under overdamped situation with no external load torque. The details of this dependence require separate study and may be discussed in future, but it is possible to discuss it for a simple case with $T(\dot{\psi}) = T_0 - \varsigma\dot{\psi}$, where $T_0 = T(0)$ and $\varsigma(> 0)$ is the sensitivity of the torque to the rotational speed. (Although the more complicated function such as $T(\dot{\psi}) \sim T_0 e^{-\dot{\psi}/\varsigma}$ would be more accurate, this makes it difficult to solve.) Then, the stational rotational angular speed becomes, $\dot{\psi} = \frac{T_0}{\gamma+\varsigma} < \frac{T_0}{\gamma}$. Therefore, it should be noted that this model, which assumes constant T, may slightly overestimate the steady-state speed. In other words, since $\dot{\psi}$ and T_0 are directly measured experimentally, the friction coefficient γ estimated using these as in the main text may include both NLCEs' viscous friction and the speed-dependent torque reduction effect. Furthermore, if there were no viscoelastic friction in this system, the rotational speed of this system would be determined by the effect of this ς; $\dot{\psi} = \frac{T_0}{\varsigma}$. This is analogous to the effect of counter-electromotive force (based on electromagnetic induction), which fundamentally determines the rotational speed of an electromagnetic motor under no load.

Factors preventing steady rotation. The above-mentioned fluctuations in the net torque with the small number of NLCE strips may cause the engine to hang up, when the lower limit becomes close to T_{load} or small static friction torque required at the start of rotation. Moreover, all NLCE strips should be installed in the same accurate manner as pointed out by Mullen for a rubber engine [2-a]. When creating an engine, it is technically important to ensure that the initial elongation given when installing the NLCE strip is as consistent as possible for all NLCE strips. Otherwise, any imbalance causes further unexpected fluctuations in the net torque. In some cases, the rotational speed fluctuations caused by this imbalance may be more pronounced than the net torque fluctuations due to the small value of *N* mentioned above, and as will be pointed out in the experimental results section, this is indeed observed in actual experiments.

Furthermore, since unevenness in the thickness of the black coating causes variation in each NLCE's response to light, it is desirable to minimize it as much as possible.

[2-a] Mullen, J. G., Wasserstein, R. & Burmeister, L. On optimizing an Archibald rubber-band heat engine. *Am. J. Phys.* **46**, 1107–1110 (1978).

Supplemental Note 3. Theoretical model on energy efficiency.

Energy efficiency. The power flowing into the mechanical system, which is the work per unit time done by the driving net torque, is expressed as $P = T\dot{\psi}$. The part of the power is converted into the available mechanical power P_{load} and the rest is dissipated as internal friction within the system P_{dis}. Here the energy conservation equation for the mechanical engine system is $P = P_{\mathrm{load}} + P_{\mathrm{dis}}$.

The available mechanical work per unit time upon stationary rotation, i.e., available power output, is calculated as $P_{\mathrm{load}} = T_{\mathrm{load}} \cdot \dot{\psi}_s = (T \cdot T_{load} - T_{load}{}^2)/\gamma$. Thus, the maximum available power output is obtained at $T_{\mathrm{load}}^* = T/2$ as $P_{\mathrm{load,max}} = T^2/4\gamma$ and the associated angular velocity $\dot{\psi}^* = (T - T_{\mathrm{load}}^*)/\gamma = T/2\gamma$. The dissipation power is expressed as $P_{\mathrm{dis}} = \gamma\dot{\psi}^2 \sim (friction\ torque) \cdot \dot{\psi}$, which is the internal frictional loss per unit time, dissipating as unusable thermal energy. The energy efficiency of the mechanical system, which is not that of the total system, becomes $\eta = P_{\mathrm{load}}/P = T_{\mathrm{load}}/T$. Thus, the efficiency at the maximum available power output becomes; $\eta_{\mathrm{max.power}} = (T/2)/T = 0.5 = 50\%$.

Assuming that the light irradiation power is the total input power P_{in}, the portion of the light power P_{loss} that is not used to raise and hold the temperature of the NLCE is dissipated by reflection or radiation from the NLCE. The total energy conservation equation becomes $P_{\mathrm{in}} = P + P_{\mathrm{loss}}$. This is rewritten as $P_{\mathrm{in}} = P_{\mathrm{load}} + P_{\mathrm{dis}} + P_{\mathrm{loss}} = T_{\mathrm{load}} \cdot \dot{\psi} + \gamma\dot{\psi}^2 + P_{\mathrm{loss}}$. Then, the total energy efficiency in the practical total engine operation is expressed as $\eta_{\mathrm{tot}} = P_{\mathrm{load}}/P_{\mathrm{in}} =$

$T_{\mathrm{load}} \cdot \dot{\psi}/P_{\mathrm{in}} = T_{\mathrm{load}} \cdot (T - T_{\mathrm{load}})/(\gamma P_{\mathrm{in}})$. The total energy efficiency at maximum power output becomes $\eta_{\mathrm{tot,maxpower}} = T^2/4\gamma P_{\mathrm{in}}$. Experimentally, P_{in} is characterized by the light power and the exposed area of NLCEs. Since other parameters, T and γ can experimentally be determined, the energy efficiency of the present system is eventually estimated as a function of T_{load}.

The upper limit of this η_{tot} is the Carnot efficiency, $\eta_{\mathrm{c}} = (\Theta_H - \Theta_L)/\Theta_H$, where temperatures in Kelvin unit. It is interesting to estimate this for a typical case with $\Theta_H \sim 360$ K, $\Theta_L \sim 330$ K, then $\eta_{\mathrm{c}} \sim 0.083 \sim 8.3\%$. Based on the above equation, the difference between η determined in the experiments described later and η_{c} originates from $P_{\mathrm{dis}} + P_{\mathrm{loss}}$.

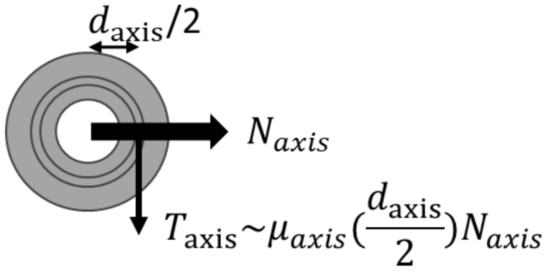

Fig. S5. Effects of mechanical parts, ball bearings, other than NLCE on rotational friction. In this mechanical system, other than the viscoelasticity of NLCE, the only source of rotational resistance is ball bearings. This resistance is incorporated into the equation of motion as a resistive torque, $I\ddot{\psi} + \gamma\dot{\psi} = T - T_{\mathrm{load}} - T_{\mathrm{axis}}$, imparting a deceleration effect on the motion analogous to T_{load}. Here, there are two bearings: one between the wheel and the main shaft, and another between the central NLCE clamping part and the offset shaft. Let the diameter of both shafts be d_{axis}. The force acting perpendicular to the shaft is approximately maximum at $\psi \sim \pi/2$ for a given opposing NLCE pair, and $\Delta f = f_H - f_L$. Thus, even if the force acting on one shaft in the $\psi \sim \pi/2$ direction is large, $N_{axis} = \Delta f N/2$. The friction torque of a single bearing is generally given by $\mu_{axis}(d_{\mathrm{axis}}/2)N_{axis}$, where μ_{axis} is the effective friction coefficient of the

ball bearing. Then, the friction torque T_{axis} resisting rotational motion of the entire assembly ($\dot{\psi} \neq 0$) incorporates the friction torque of two bearings, $T_{\text{axis}} = 2\mu_{axis}(d_{\text{axis}}/2)N_{axis} = 2\mu_{axis}(d_{\text{axis}}/2)\Delta f(N/2) = \mu_{axis}(d_{\text{axis}}/2)N\Delta f$. We wish to consider how much this shaft friction might impede rotational motion, so we compare it with the drive torque. The drive torque is given by $T = r\ N\Delta f/\pi$, thus, we only need to compare $\mu_{axis}d_{\text{axis}}/2$ with r/π . Here, d_{axis} ~ 2.95 mm, and r = 25.9 mm, yielding, $\mu_{axis}d_{\text{axis}}/2{\sim}1.475\mu_{axis}$ and $r/\pi{\sim}8.244$. Here, μ_{axis} is generally small for ball bearings, typically less than 0.01 [4-a]. For example, inserting this value yields: $\mu_{axis}d_{\text{axis}}/2$ ~0.01475≪8.244, ultimately yielding $T_{\text{axis}} \ll T$. Thus, T_{axis} may be neglected. Ultimately, the present primary internal resistance can be considered to be the viscous resistance γ originating from NLCE.

[4-a] P. Wu, C. He, G. Chen, C. Ren, Determination of the equivalent friction coefficient of rolling bearings using the kinetic energy dissipation, *Measurement* **244**, 2025, 116533, https://doi.org/10.1016/j.measurement.2024.116533

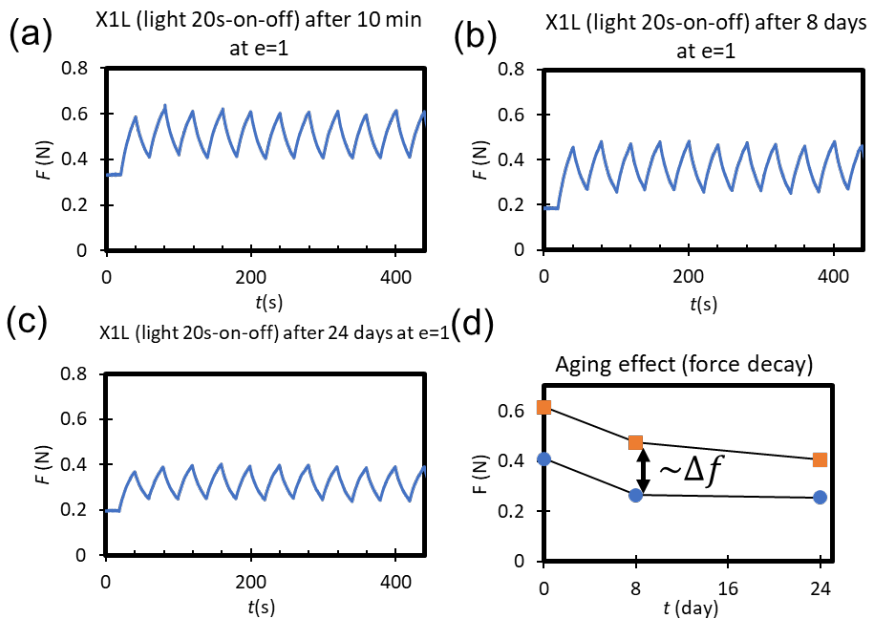

Fig. S6. Long-term light-induced force decay and expected engine fatigue. Force change of the present NLCE strip at a constant strain (e =1) in response to 20 s on/off cycles of constant-power light irradiation at (a) 1 min, (b) 8 days, and (c) 24 days after applying strain e =1. (e) The decay of average force change in (a)-(c) over time. Under long-term aging at e =1 (tensile), the force change Δf decreases by approximately 10% after 8 days and approximately 20% after 24 days. Therefore, considering continuous engine operation, Δf required for engine rotation may eventually decrease after long-term strain cycling, potentially engine rotation may stop. In such a case, reapplying pre-tension for adjustment should restore engine rotation capability. As long as there is no irreversible fatigue in the NLCE's polymer cross-linked structure, it should remain operational. Under conditions of significantly strong irradiation, we have observed that movement can continue for up to several hours. However, when starting under irradiation conditions that barely allow movement, there were cases where movement ceased within minutes. Therefore, the effect of long-term stress relaxation under tension can manifest as cessation of movement depending on the operating conditions. If we could create a cross-linked polymer network that resists this stress relaxation, we might be able to reduce this fatigue, but this remains a future challenge.
